# Supplementary figures and images for: NCBP2 modulates neurodevelopmental defects of the 3q29 deletion in Drosophila and Xenopus laevis models
Source: PLoS Genet. 2020 Feb 13;16(2):e1008590. doi: 10.1371/journal.pgen.1008590 (PMC7043793; doi:10.1371/journal.pgen.1008590)

**A****Adult wing defects**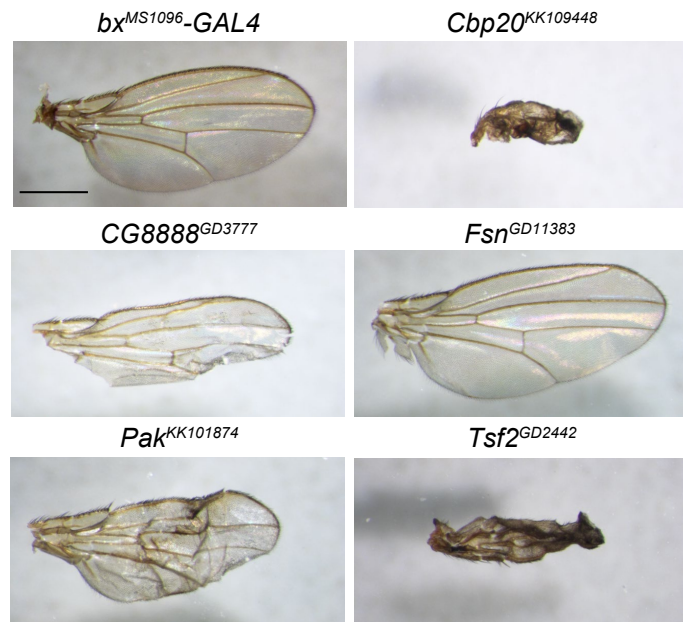**B****Survival assay**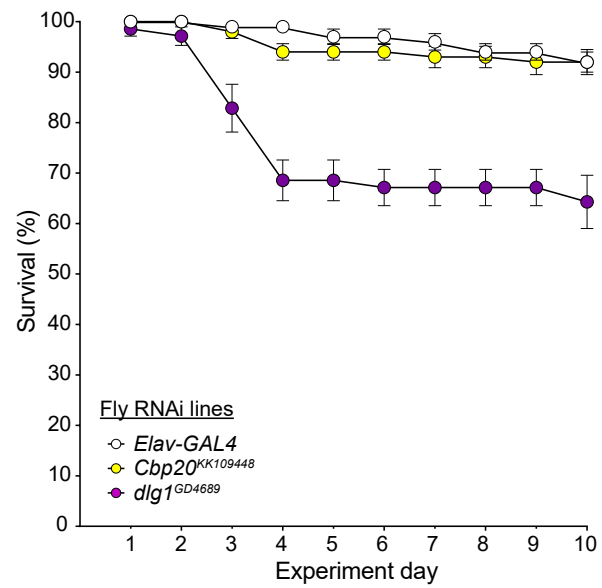**C****Axon targeting defects**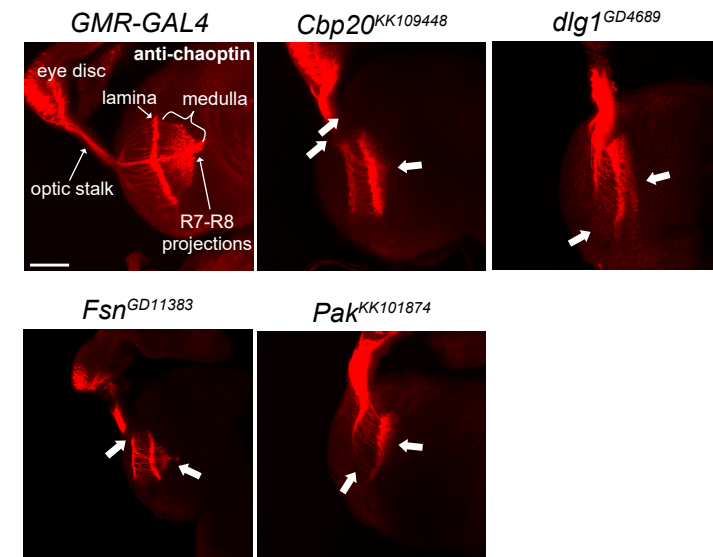

Supplement: S1 Fig — (A) Images of adult fly wings (scale bar = 500um) show a range of phenotypic defects due to wing-specific bxMS1096-GAL4 RNAi knockdown of fly homologs of 3q29 genes. (B) Adult flies with pan-neuronal RNAi knockdown of dlg1 showed approximately 30% lethality between days 1–4 (one-way repeated measures ANOVA, p<1×10^−4^, df = 1, F = 54.230), which was not observed in control Elav-GAL4 or Cbp20 knockdown flies. Data represented shows mean ± standard deviation of 10 independent groups of 10 flies for each homolog. (C) Representative confocal images of larval eye discs stained with anti-chaoptin (scale bar = 30 μm) illustrate defects in axon targeting (highlighted by white arrows) from the retina to the optic lobes of the brain upon eye-specific knockdown of fly homologs of 3q29 genes. Note that n = 8–20 larval eye disc preparations were assessed for each RNAi line tested. A list of full genotypes for fly crosses used in these experiments is provided in S2 File. (PDF) [file pgen.1008590.s001.pdf]

**A**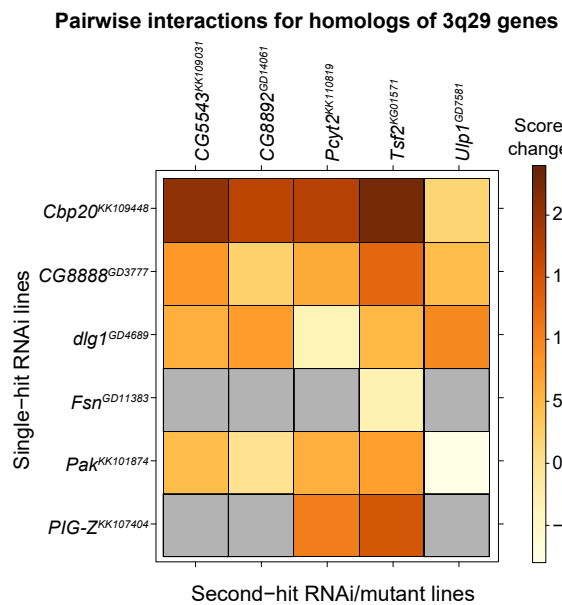**B**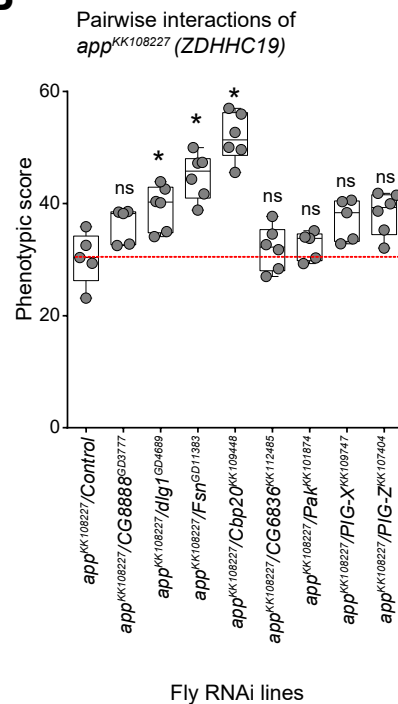**C**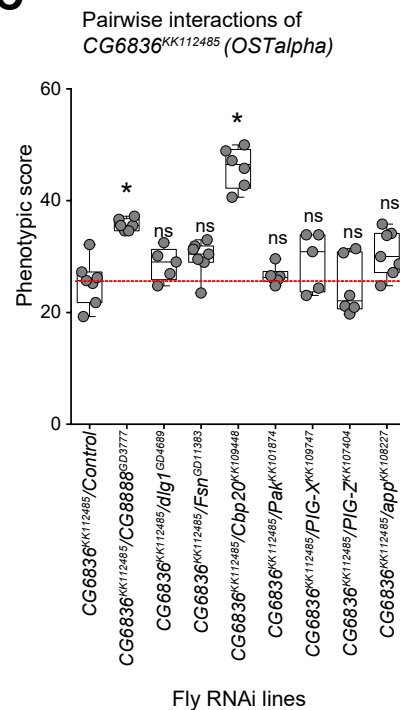**D**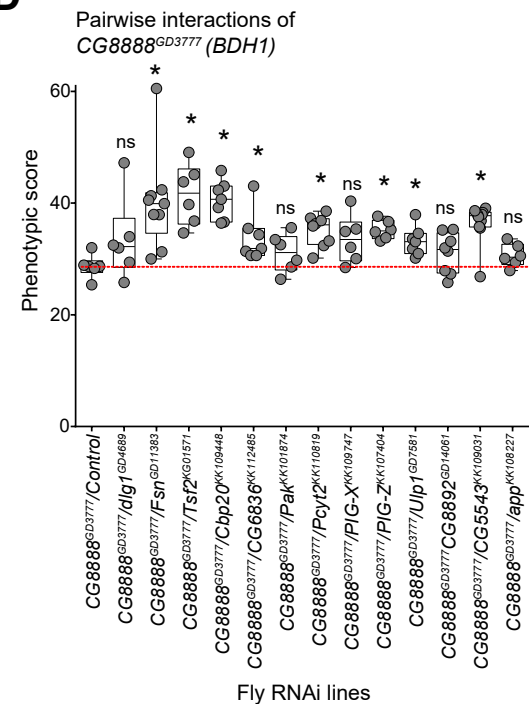**E**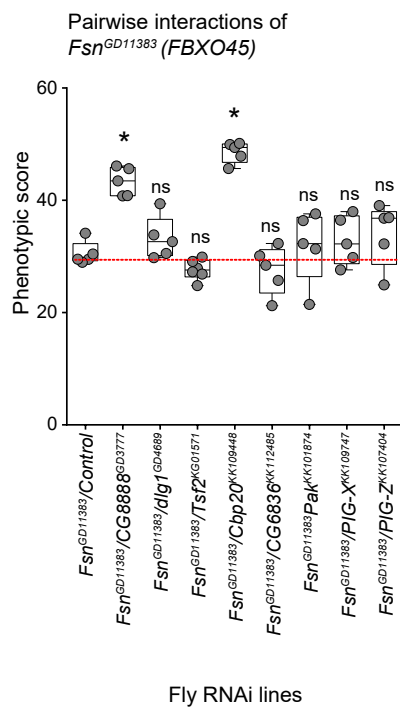**F**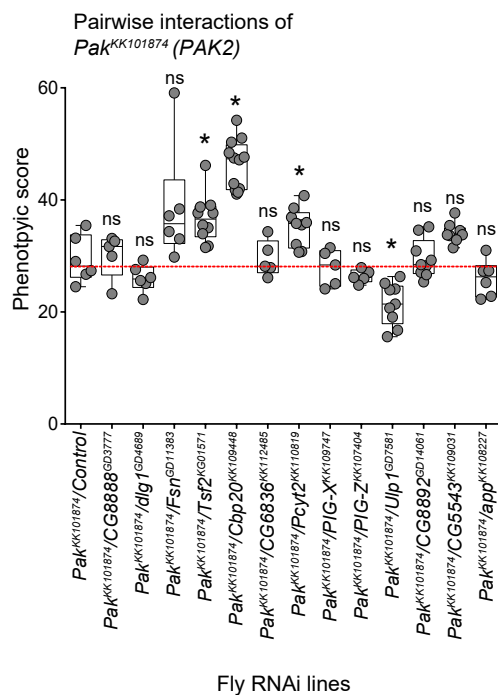**G**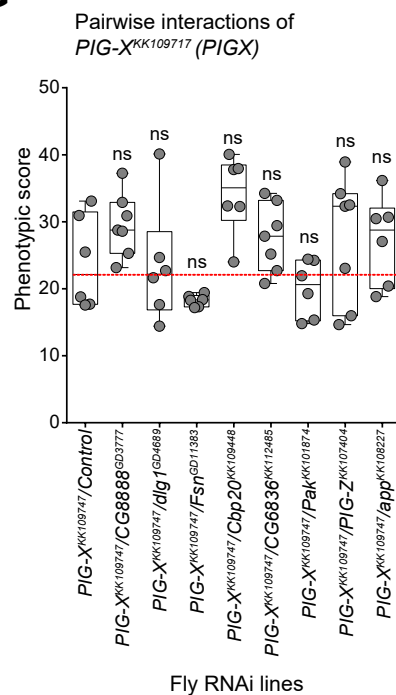**H**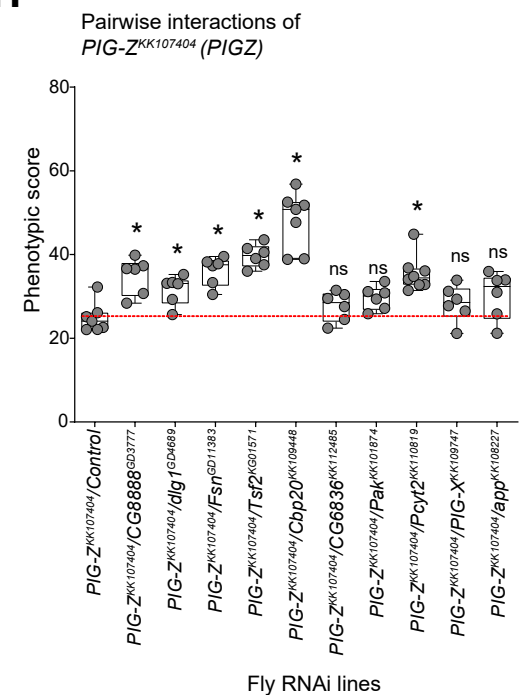

Supplement: S6 Fig — (A) Heatmap showing average changes in phenotypic scores for pairwise GMR-GAL4 RNAi knockdown of fly homologs of 3q29 genes in the adult eye, compared with recombined lines for individual homologs of 3q29 genes crossed with controls, is shown. Gray boxes indicate crosses without available data. Crosses with the mutant line Tsf2KG01571 are also included along with RNAi lines for other homologs of 3q29 genes, as eye-specific RNAi knockdown of Tsf2 was lethal. (B-H) Box plots of phenotypic scores for pairwise knockdowns of homologs of 3q29 genes compared with recombined lines for individual homologs of 3q29 genes crossed with controls are shown (n = 5–12, *p < 0.05, two-tailed Mann–Whitney test with Benjamini-Hochberg correction). All boxplots indicate median (center line), 25th and 75th percentiles (bounds of box), and minimum and maximum (whiskers), with red dotted lines representing the control median. A list of full genotypes for fly crosses used in these experiments is provided in S2 File. (PDF) [file pgen.1008590.s006.pdf]

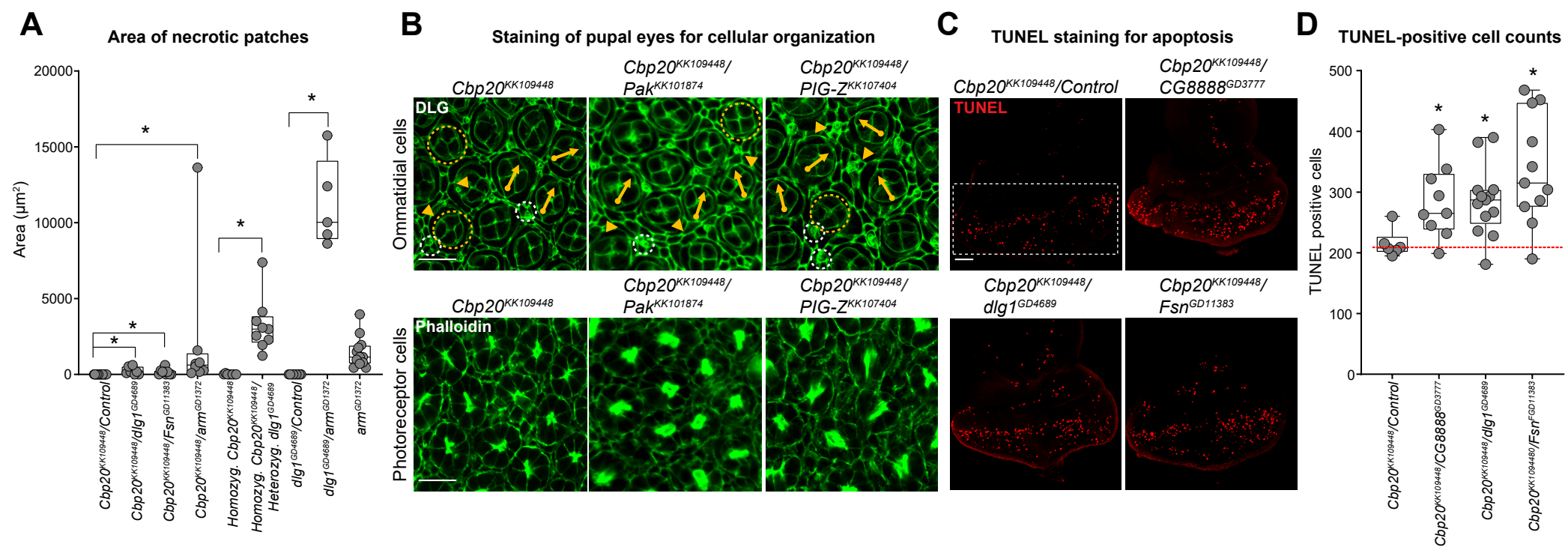

Supplement: S9 Fig — (A) Box plot showing the area of necrotic patches in adult fly eyes with pairwise knockdown of homologs of 3q29 genes (n = 5–13, *p < 0.05, one-tailed Mann–Whitney test with Benjamini-Hochberg correction). Flies with knockdown of Cbp20 and Fsn, dlg1 and arm showed enhanced necrotic patches compared with knockdown of Cbp20, while homozygous Cbp20 RNAi and concomitant knockdown of dlg1 showed increased necrotic patches compared with homozygous Cbp20 RNAi. Furthermore, flies with knockdown of dlg1 and arm both showed enhanced necrotic patches compared with individual knockdown of dlg1 or arm. (B) Confocal images of pupal eyes (scale bar = 5 μm) stained with anti-DLG (top) and Phalloidin (bottom) illustrate enhanced defects in ommatidial and photoreceptor cell organization with concomitant GMR-GAL4 RNAi knockdown of Cbp20 and other fly homologs of 3q29 genes compared with Cbp20 knockdown. (C) Larval eye discs (scale bar = 30 μm) stained with TUNEL show increases in apoptosis with pairwise knockdown of Cbp20 and other fly homologs of 3q29 genes compared with recombined Cbp20 knockdown crossed with control. (D) Box plot of TUNEL-positive cells in the larval eye discs of flies with pairwise knockdown of homologs of 3q29 genes (n = 9–13, *p < 0.05, two-tailed Mann–Whitney test with Benjamini-Hochberg correction). All boxplots indicate median (center line), 25th and 75th percentiles (bounds of box), and minimum and maximum (whiskers), with red dotted lines representing the control median. A list of full genotypes for fly crosses used in these experiments is provided in S2 File. (PDF) [file pgen.1008590.s009.pdf]

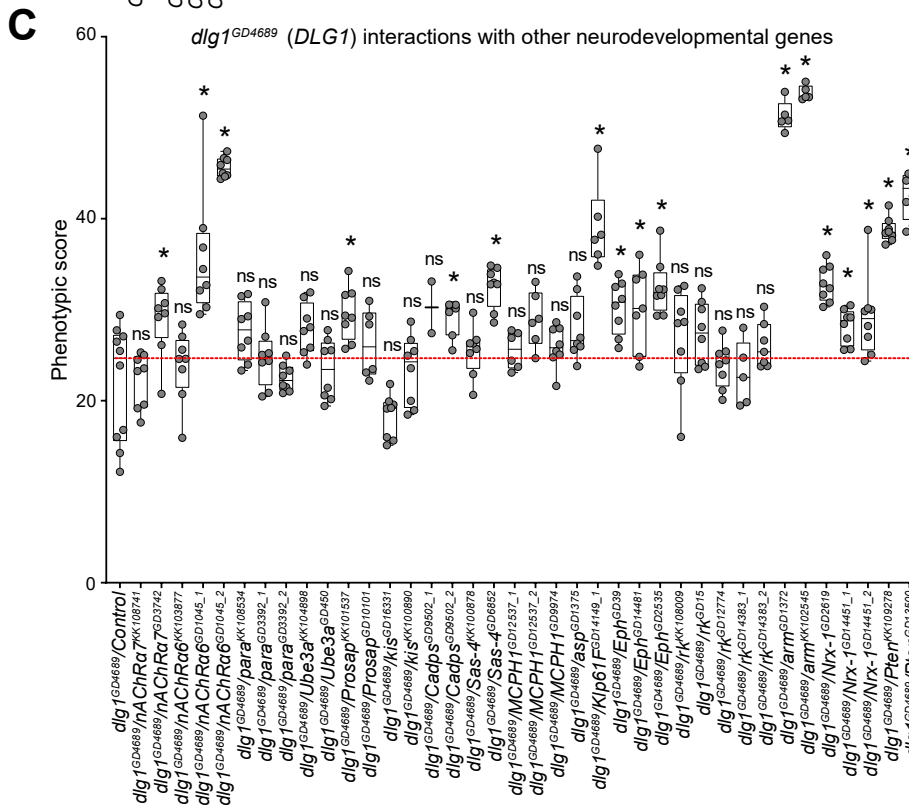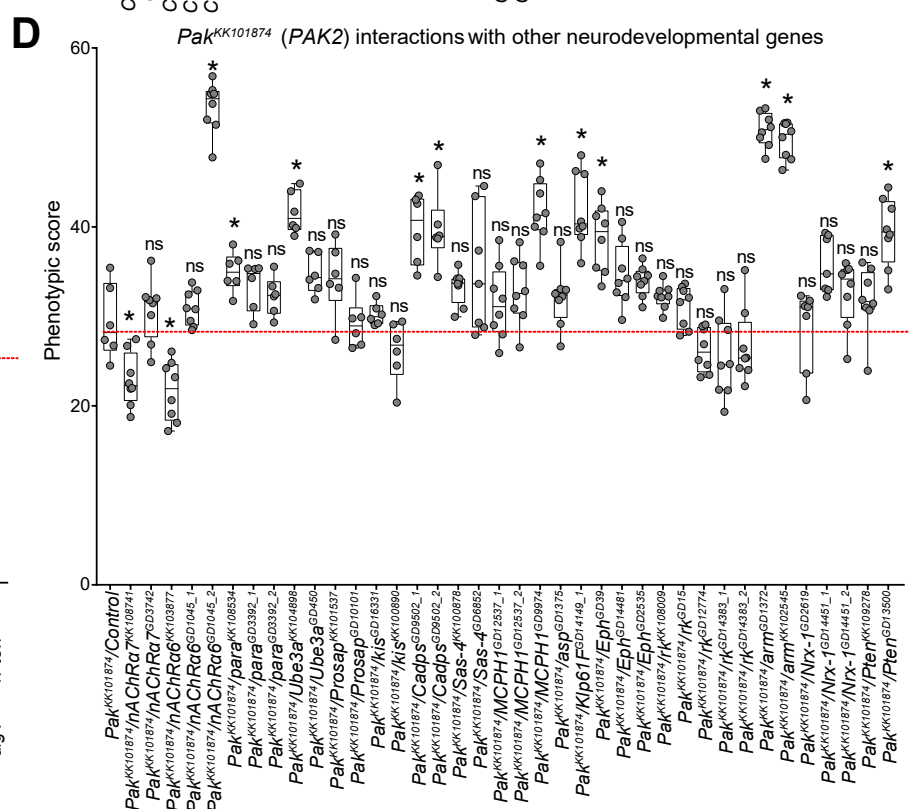

Supplement: S11 Fig — (A-D) Box plots of phenotypic scores for concomitant GMR-GAL4 RNAi knockdown of fly homologs of 3q29 genes and neurodevelopmental genes, compared with recombined lines for individual homologs of 3q29 genes crossed with controls, are shown (n = 2–10, *p < 0.05, two-tailed Mann–Whitney test with Benjamini-Hochberg correction). All boxplots indicate median (center line), 25th and 75th percentiles (bounds of box), and minimum and maximum (whiskers), with red dotted lines representing the control median. A list of full genotypes for fly crosses used in these experiments is provided in S2 File. (PDF) [file pgen.1008590.s011.pdf]

**A**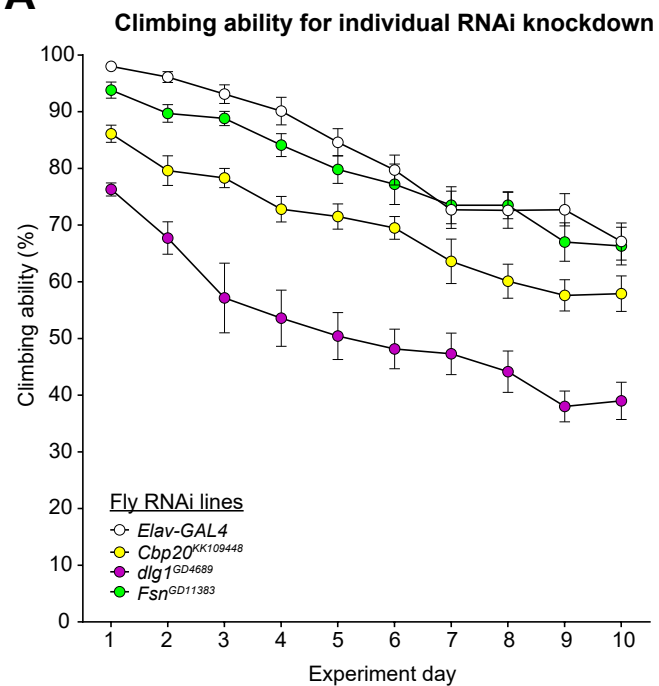**B**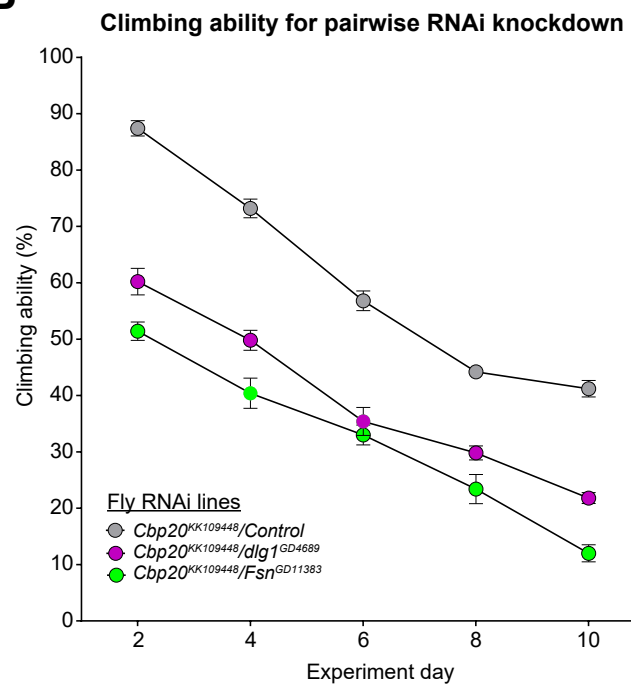**C**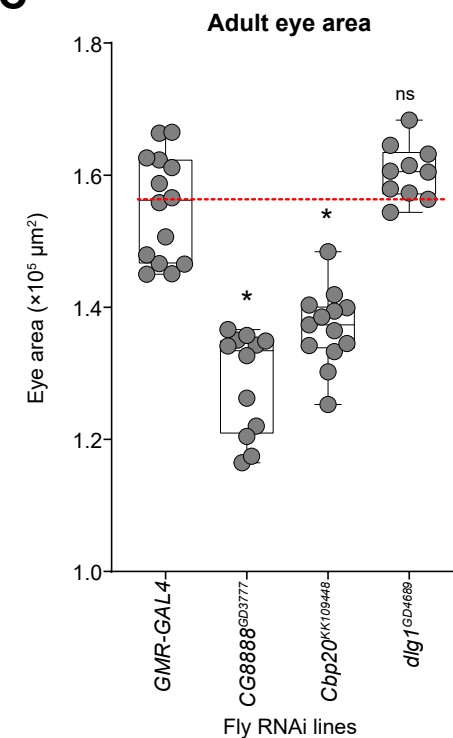**D**

**Cellular phenotypes in the larval eye disc**

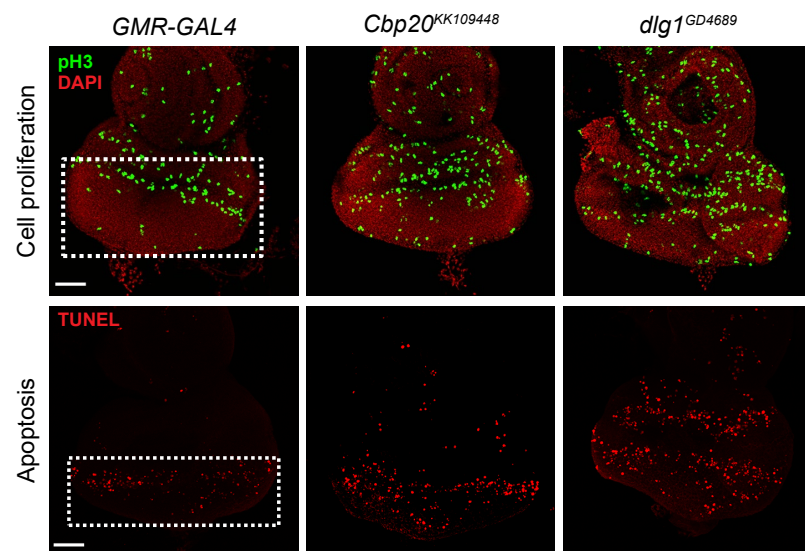**E**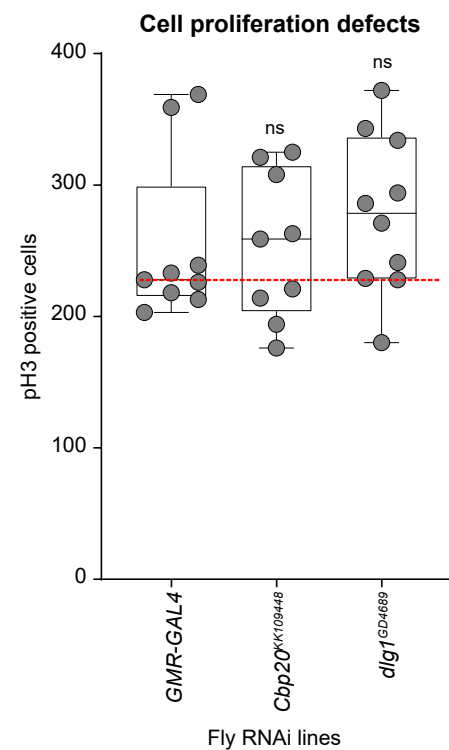**F**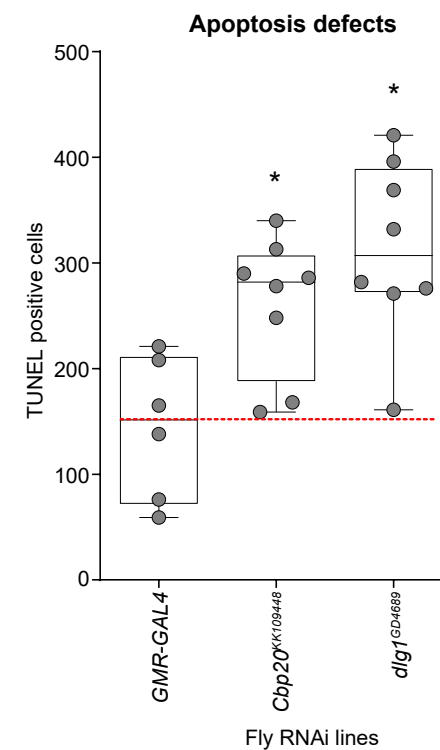

Supplement: S14 Fig — (A) Replication dataset for climbing ability of select homologs of 3q29 genes over ten days. We replicated the defects in climbing ability observed with pan-neuronal RNAi knockdown of Cbp20 and dlg1, while climbing defects in flies with knockdown of Fsn flies were not replicated in the second experimental batch and were therefore excluded from the main dataset (Fig 2B). Data represented show mean ± standard deviation of 7–10 independent groups of 10 flies for each homolog. (B) Replication dataset for climbing ability of pairwise knockdown of homologs of 3q29 genes over ten days. We replicated the defects in climbing ability observed with pan-neuronal RNAi knockdown of Cbp20/dlg1 and Cbp20/Fsn compared with recombined Cbp20 knockdown crossed with control (Fig 3F). Data represented show mean ± standard deviation of 5 independent groups of 10 flies for each homolog. (C) Replication dataset for adult eye area in flies with GMR-GAL4 RNAi knockdown of homologs of 3q29 genes (n = 10–14, *p < 0.05, two-tailed Mann–Whitney test with Benjamini-Hochberg correction). We replicated the decreased eye sizes in flies with knockdown of Cbp20 and CG8888, while flies with knockdown of dlg1 showed a non-significant (p = 0.154) increase in eye size (Fig 2D). (D) Confocal images for replication dataset larval eye discs (scale bar = 30 μm) stained with anti-pH3 (top) and TUNEL (bottom) illustrate cellular defects posterior to the morphogenetic furrow (white box) upon knockdown of select fly homologs of 3q29 genes (Fig 2E). (E) Replication dataset for pH3-positive cells in larval eye discs of flies with knockdown of homologs of 3q29 genes (n = 9–10, two-tailed Mann–Whitney test with Benjamini-Hochberg correction). As in the main dataset (Fig 2F), we observed no significant changes in cell proliferation for flies with knockdown of Cbp20 and dlg1. (F) Replication dataset for TUNEL-positive cells in larval eye discs of flies with knockdown of homologs of 3q29 genes (n = 6–8, *p < 0.05, two-ta [file pgen.1008590.s014.pdf]
