# Supplementary material for: NCBP2 modulates neurodevelopmental defects of the 3q29 deletion in Drosophila and Xenopus laevis models
Source: PLoS Genet. 2020 Feb 13;16(2):e1008590. doi: 10.1371/journal.pgen.1008590 (PMC7043793; doi:10.1371/journal.pgen.1008590)
Supplement: S2 Fig — We tested individual and pairwise knockdown of fly homologs of 3q29 genes for cellular phenotypes in the adult, pupal and larval eyes. (A) We first used the Flynotyper software [53] to quantify the degree of ommatidial disorganization leading to rough eye phenotypes in adult flies, as represented by the distance and angles between adjacent ommatidia (yellow arrows). (B) We next stained pupal eyes with anti-DLG to observe changes in the number and arrangement of ommatidial cells, including cone cells (c), bristle cells (b), and primary, secondary and tertiary cells (1,2,3). We also examined the organization of the photoreceptor cells (R1-R7, with R8 not visible) in each ommatidium by staining the pupal eyes with Phalloidin. (C) We finally stained larval eye discs with markers for cellular processes, such as pH3 for proliferating cells and dcp1 for apoptosis. As the progression of the morphogenetic furrow (MF) across the larval eye discs leads to proliferation and differentiation of photoreceptor neurons [121], we examined changes in the number of stained cells posterior to the MF. (D) Scatter plot of dcp1, pH3, TUNEL, and BrdU-positive cell counts in larval eye discs with knockdown of homologs of 3q29 genes quantified using two ImageJ plugins, AnalyzeParticles and Image-based Tool for Counting Nuclei (ITCN). As the two methods showed a strong correlation with each other (Pearson correlation, n = 285, r = 0.736, p<2.2×10^−16^), we used ITCN counts to display cell count data in the manuscript. (PDF) [file pgen.1008590.s002.pdf]

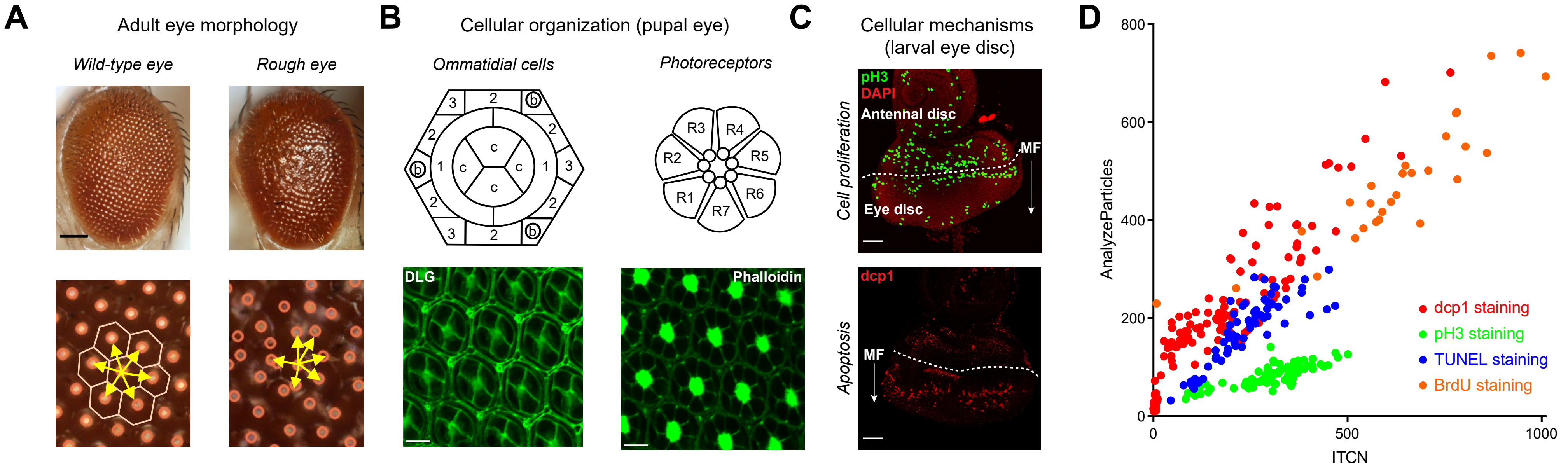
A
Adult eye morphology
Wild-type eye
Rough eye
B
Cellular organization (pupal eye)
Ommatidial cells
Photoreceptors
DLG
Phalloidin
C
Cellular mechanisms
(larval eye disc)
Cell proliferation
pH3
DAPI
Antennal disc
MF
Eye disc
Apoptosis
dcp1
MF
D
AnalyzeParticles
ITCN
dcp1 staining
pH3 staining
TUNEL staining
BrdU staining
