# Supplementary material for: NCBP2 modulates neurodevelopmental defects of the 3q29 deletion in Drosophila and Xenopus laevis models
Source: PLoS Genet. 2020 Feb 13;16(2):e1008590. doi: 10.1371/journal.pgen.1008590 (PMC7043793; doi:10.1371/journal.pgen.1008590)
Supplement: S3 Fig — (A) Representative brightfield adult eye images of flies with GMR-GAL4;UAS-Dicer2 RNAi knockdown of fly homologs of 3q29 genes (scale bar = 100 μm) show a wide range of phenotypic severity. (B) Box plot of average ommatidial diameter in flies with GMR-GAL4 knockdown of select fly homologs of 3q29 genes is shown (n = 15, *p < 0.05, two-tailed Mann–Whitney test with Benjamini-Hochberg correction). (C) Box plot of phenotypic scores derived from Flynotyper for eye-specific dCad-GFP,GMR-GAL4 RNAi knockdown of 13 fly homologs of 3q29 genes is shown (n = 5–20, *p < 0.05, one-tailed Mann–Whitney test with Benjamini-Hochberg correction). (D) Box plot of phenotypic scores derived from Flynotyper for eye-specific GMR-GAL4;UAS-Dicer2 (left) and dCad-GFP,GMR-GAL4 (right) RNAi knockdown of nine validation lines for fly homologs of 3q29 genes is shown (n = 5–14, *p < 0.05, one-tailed Mann–Whitney test with Benjamini-Hochberg correction). All boxplots indicate median (center line), 25th and 75th percentiles (bounds of box), and minimum and maximum (whiskers), with red dotted lines representing the control median. A list of full genotypes for fly crosses used in these experiments is provided in S2 File. (PDF) [file pgen.1008590.s003.pdf]

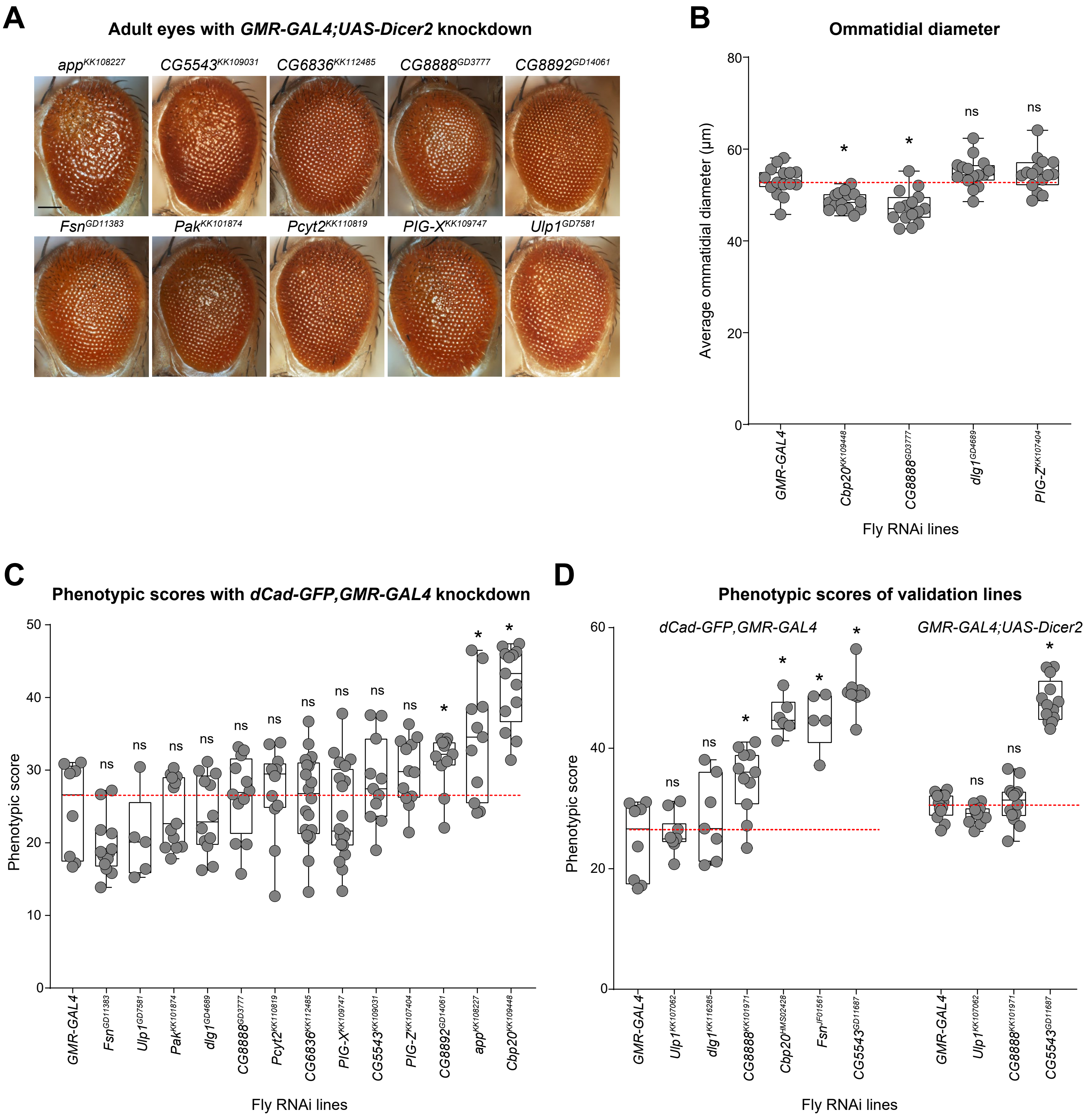
A
Adult eyes with GMR-GAL4;UAS-Dicer2 knockdown
appKK108227
CG5543KK109031
CG6836KK112485
CG8888GD3777
CG8892GD14061
FsnGD11383
PakKK101874
Pcyt2KK110819
PIG-XKK109747
Ulp1GD7581
B
Ommatidial diameter
Average ommatidial diameter (µm)
80
60
40
20
0
*
*
ns
ns
GMR-GAL4
Cbp20KK109448
CG8888GD3777
dlg1GD4689
PIG-ZKK107404
Fly RNAi lines
C
Phenotypic scores with dCad-GFP,GMR-GAL4 knockdown
Phenotypic score
50
40
30
20
10
0
ns
ns
ns
ns
ns
ns
ns
ns
ns
ns
*
*
*
GMR-GAL4
FsnGD11383
Ulp1GD7581
PakKK101874
dlg1GD4689
CG8888GD3777
Pcyt2KK110819
CG6836KK112485
PIG-XKK109747
CG5543KK109031
PIG-ZKK107404
CG8892GD14061
appKK108227
Cbp20KK109448
Fly RNAi lines
D
Phenotypic scores of validation lines
dCad-GFP,GMR-GAL4
GMR-GAL4;UAS-Dicer2
Phenotypic score
60
40
20
0
ns
ns
*
*
*
*
ns
ns
*
GMR-GAL4
Ulp1KK107062
dlg1KK116285
CG8888KK101971
Cbp20HMS02428
FsnJF01561
CG5543GD11687
GMR-GAL4
Ulp1KK107062
CG8888KK101971
CG5543GD11687
Fly RNAi lines
