# Supplementary material for: NCBP2 modulates neurodevelopmental defects of the 3q29 deletion in Drosophila and Xenopus laevis models
Source: PLoS Genet. 2020 Feb 13;16(2):e1008590. doi: 10.1371/journal.pgen.1008590 (PMC7043793; doi:10.1371/journal.pgen.1008590)
Supplement: S4 Fig — (A) Confocal images of pupal eyes (scale bar = 5 μm) stained with anti-DLG illustrate a range of defects in ommatidial organization upon GMR-GAL4 RNAi knockdown of fly homologs of 3q29 genes. Yellow circles indicate cone cell defects, white circles indicate bristle cell defects, yellow arrows indicate rotation defects, and yellow arrowheads indicate secondary cell defects. (B) Confocal images of pupal eyes (scale bar = 5 μm) stained with Phalloidin illustrate defects in photoreceptor cell count and organization upon knockdown of fly homologs of 3q29 genes. (C) Confocal images of larval eye discs (scale bar = 30 μm) stained with anti-pH3 illustrate changes in cell proliferation upon knockdown of select fly homologs of 3q29 genes. (D) Larval eye discs (scale bar = 30 μm) stained with BrdU (top) and TUNEL (bottom) illustrate abnormal cell cycle and apoptosis defects, respectively, due to eye-specific knockdown of Cbp20 and dlg1. (E) Box plot of BrdU-positive cells in the larval eye discs of flies with knockdown of dlg1 and Cbp20 is shown (n = 7–12, *p < 0.05, two-tailed Mann–Whitney test with Benjamini-Hochberg correction). (F) Box plot of TUNEL-positive cells in the larval eye discs of flies with knockdown of dlg1 and Cbp20 is shown (n = 8, *p < 0.05, two-tailed Mann–Whitney test with Benjamini-Hochberg correction). Results for the TUNEL staining experiments were replicated in an independent experimental batch (S14 Fig). All boxplots indicate median (center line), 25th and 75th percentiles (bounds of box), and minimum and maximum (whiskers), with red dotted lines representing the control median. A list of full genotypes for fly crosses used in these experiments is provided in S2 File. (PDF) [file pgen.1008590.s004.pdf]

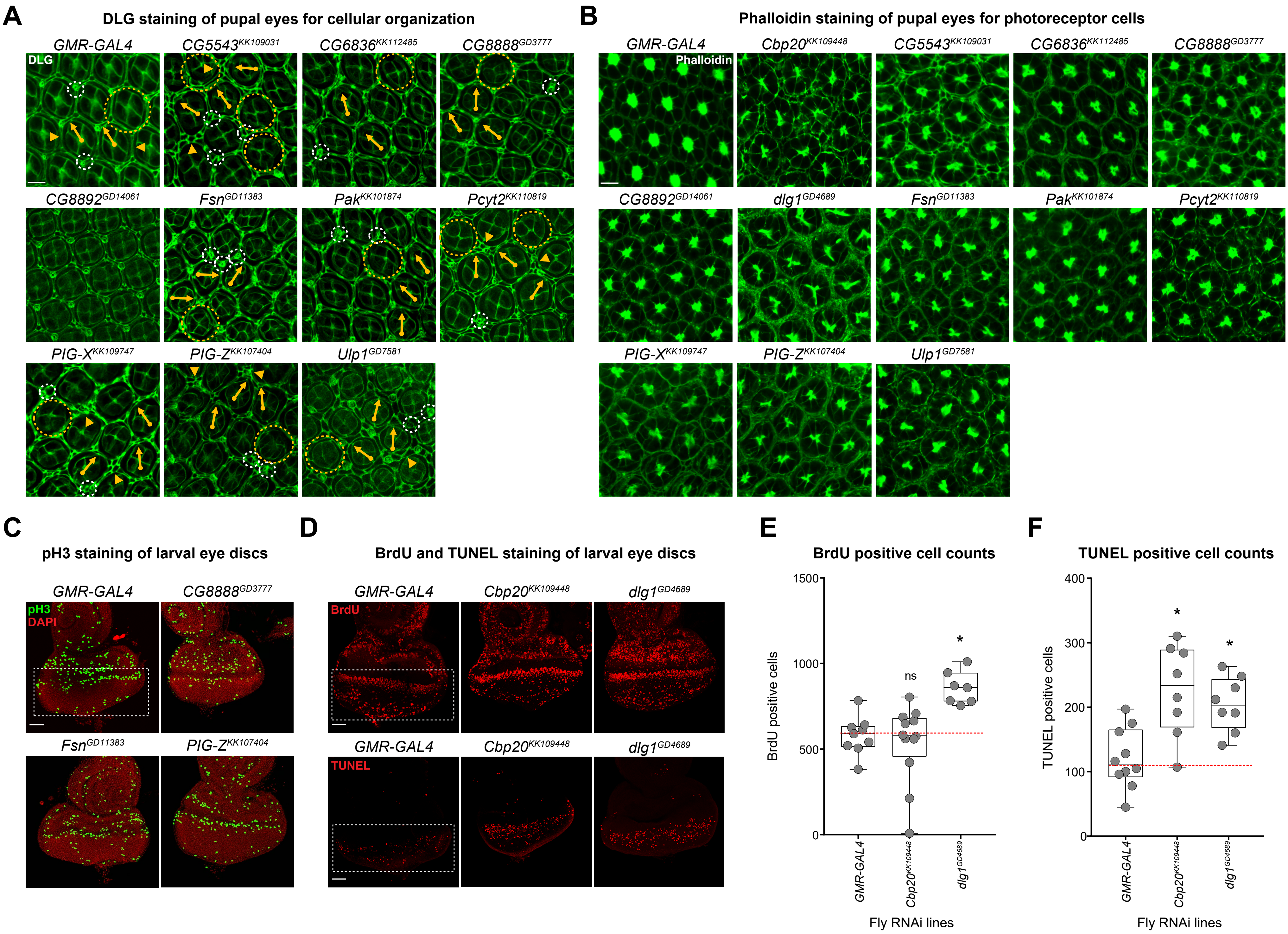

A
DLG staining of pupal eyes for cellular organization
GMR-GAL4
CG5543KK109031
CG6836KK112485
CG8888GD3777
DLG
CG8892GD14061
FsnGD11383
PakKK101874
Pcyt2KK110819
PIG-XKK109747
PIG-ZKK107404
Ulp1GD7581
B
Phalloidin staining of pupal eyes for photoreceptor cells
GMR-GAL4
Cbp20KK109448
CG5543KK109031
CG6836KK112485
CG8888GD3777
Phalloidin
CG8892GD14061
dlg1GD4689
FsnGD11383
PakKK101874
Pcyt2KK110819
PIG-XKK109747
PIG-ZKK107404
Ulp1GD7581
C
pH3 staining of larval eye discs
GMR-GAL4
CG8888GD3777
pH3
DAPI
FsnGD11383
PIG-ZKK107404
D
BrdU and TUNEL staining of larval eye discs
GMR-GAL4
Cbp20KK109448
dlg1GD4689
BrdU
TUNEL
E
BrdU positive cell counts
BrdU positive cells
1500
1000
500
0
ns
*
GMR-GAL4
Cbp20KK109448
dlg1GD4689
Fly RNAi lines
F
TUNEL positive cell counts
TUNEL positive cells
400
300
200
100
0
*
*
GMR-GAL4
Cbp20KK109448
dlg1GD4689
Fly RNAi lines
