# Supplementary material for: NCBP2 modulates neurodevelopmental defects of the 3q29 deletion in Drosophila and Xenopus laevis models
Source: PLoS Genet. 2020 Feb 13;16(2):e1008590. doi: 10.1371/journal.pgen.1008590 (PMC7043793; doi:10.1371/journal.pgen.1008590)
Supplement: S5 Fig — (A) Larval wing discs (scale bar = 50 μm) stained with pH3 illustrate abnormal cell proliferation due to RNAi knockdown of select fly homologs of 3q29 genes, compared with appropriate VDRC GD and KK bxMS1096-GAL4 controls. We examined changes in the number of stained cells within the wing pouch of the wing disc (white box), which becomes the adult wing. (B) Box plot of pH3-positive cells in the larval wing discs of flies with knockdown of select fly homologs of 3q29 genes is shown (n = 8–15, *p < 0.05, two-tailed Mann–Whitney test with Benjamini-Hochberg correction). (C) Larval wing discs (scale bar = 50 μm) stained with anti-dcp1 show abnormal apoptosis due to knockdown of select fly homologs of 3q29 genes compared with appropriate VDRC GD and KK bxMS1096-GAL4 controls. (D) Box plot of dcp1-positive cells in the larval wing discs of flies with knockdown of select fly homologs of 3q29 genes is shown (n = 8–15, *p < 0.05, two-tailed Mann–Whitney test with Benjamini-Hochberg correction). Cbp20 flies showed severe dcp1 staining across the entire wing pouch and could not be quantified. All boxplots indicate median (center line), 25th and 75th percentiles (bounds of box), and minimum and maximum (whiskers), with red dotted lines representing the control median. A list of full genotypes for fly crosses used in these experiments is provided in S2 File. (PDF) [file pgen.1008590.s005.pdf]

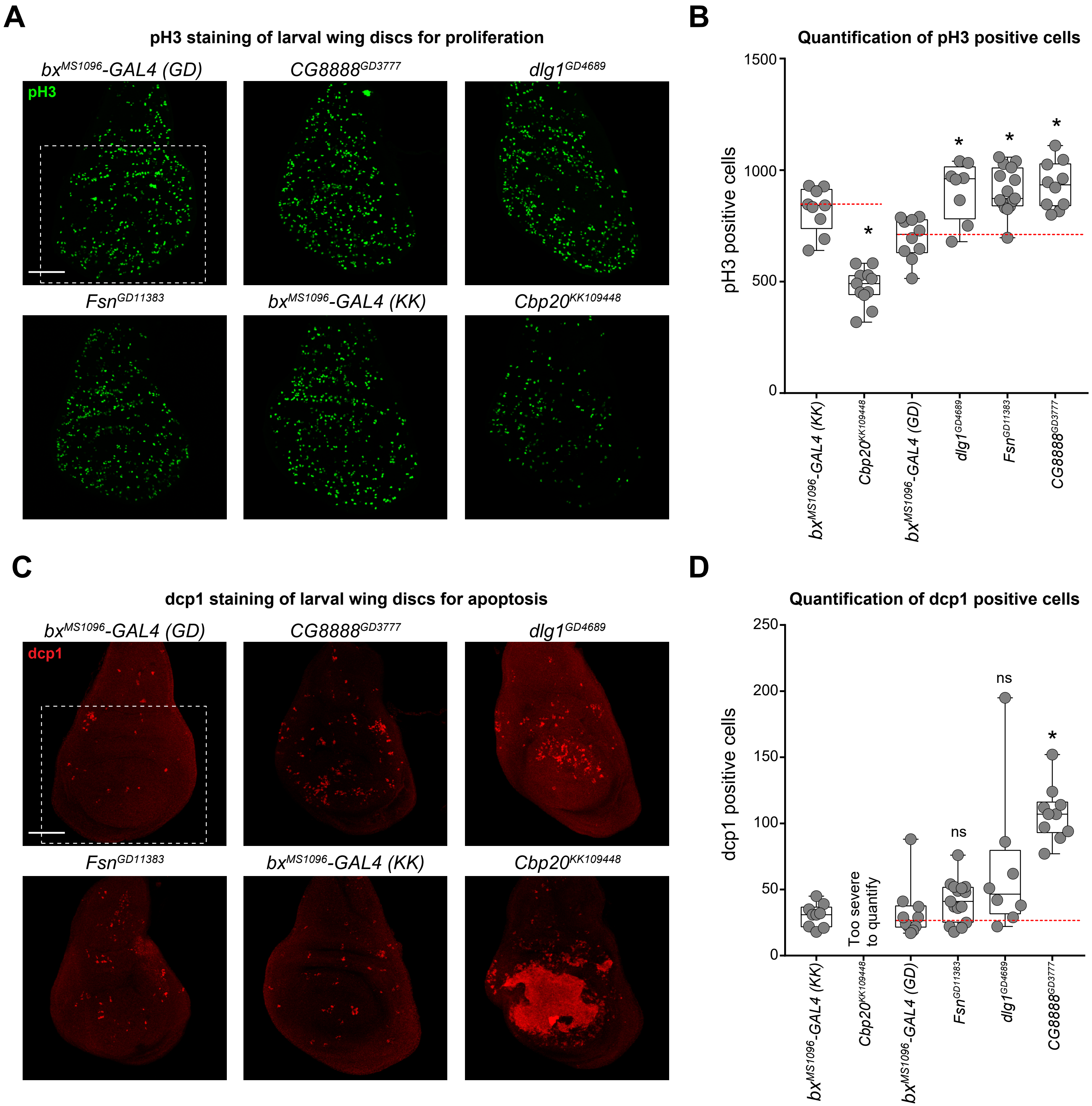

A
pH3 staining of larval wing discs for proliferation
bxMS1096-GAL4 (GD)
CG8888GD3777
dlg1GD4689
pH3
FsnGD11383
bxMS1096-GAL4 (KK)
Cbp20KK109448
B
Quantification of pH3 positive cells
pH3 positive cells
1500
1000
500
0
*
bxMS1096-GAL4 (KK)
Cbp20KK109448
bxMS1096-GAL4 (GD)
dlg1GD4689
FsnGD11383
CG8888GD3777
C
dcp1 staining of larval wing discs for apoptosis
bxMS1096-GAL4 (GD)
CG8888GD3777
dlg1GD4689
dcp1
FsnGD11383
bxMS1096-GAL4 (KK)
Cbp20KK109448
D
Quantification of dcp1 positive cells
dcp1 positive cells
250
200
150
100
50
0
ns
*
Too severe to quantify
bxMS1096-GAL4 (KK)
Cbp20KK109448
bxMS1096-GAL4 (GD)
FsnGD11383
dlg1GD4689
CG8888GD3777
