# Supplementary material for: NCBP2 modulates neurodevelopmental defects of the 3q29 deletion in Drosophila and Xenopus laevis models
Source: PLoS Genet. 2020 Feb 13;16(2):e1008590. doi: 10.1371/journal.pgen.1008590 (PMC7043793; doi:10.1371/journal.pgen.1008590)
Supplement: S8 Fig — (A) Clusters of Gene Ontology terms enriched among differentially-expressed fly genes (blue) and their corresponding human homologs (red) with individual and pairwise Elav-GAL4 RNAi knockdown of fly homologs of 3q29 genes (p< 0.05, Fisher’s Exact test with Benjamini-Hochberg correction) are shown. Black boxes indicate enrichment of each gene set for clusters of Gene Ontology terms. Full lists of enriched GO terms are provided in S3 File. (B) Enrichments for shared and unique differentially-expressed fly genes (blue) and their corresponding human homologs (red) with individual knockdown of Cbp20 and Fsn, as well as concomitant knockdown of Cbp20/Fsn, are shown. We found 229 genes uniquely dysregulated in flies with pairwise knockdown of Fsn and Cbp20, which were enriched for cell cycle function (p = 0.011 for fly gene enrichment and p = 1.12×10^−8^ for human homologs, Fisher’s Exact test with Benjamini-Hochberg correction). (C) Diagram showing human cell cycle and apoptosis genes whose fly homologs are differentially expressed with knockdown of Cbp20 and Fsn, as well as concomitant knockdown of Cbp20/Fsn. Red boxes indicate apoptosis genes, green boxes indicate cell cycle genes, and yellow boxes indicate genes associated with both functions. (D) Enrichments of human homologs of genes differentially expressed in flies with knockdown of Cbp20/Fsn across different brain tissues and developmental timepoints are shown (Specific Expression Analysis). The size of each hexagon represents the number of genes preferentially expressed at each tissue and timepoint, with concentric hexagons representing bins of genes with stronger levels of preferential expression. The shading of each hexagon represents the enrichment of differentially-expressed genes among genes preferentially expressed at each timepoint (p<0.1, Fisher’s Exact test with Benjamini-Hochberg correction). A list of full genotypes for fly crosses used in these experiments is provided in S2 File. (PDF) [file pgen.1008590.s008.pdf]

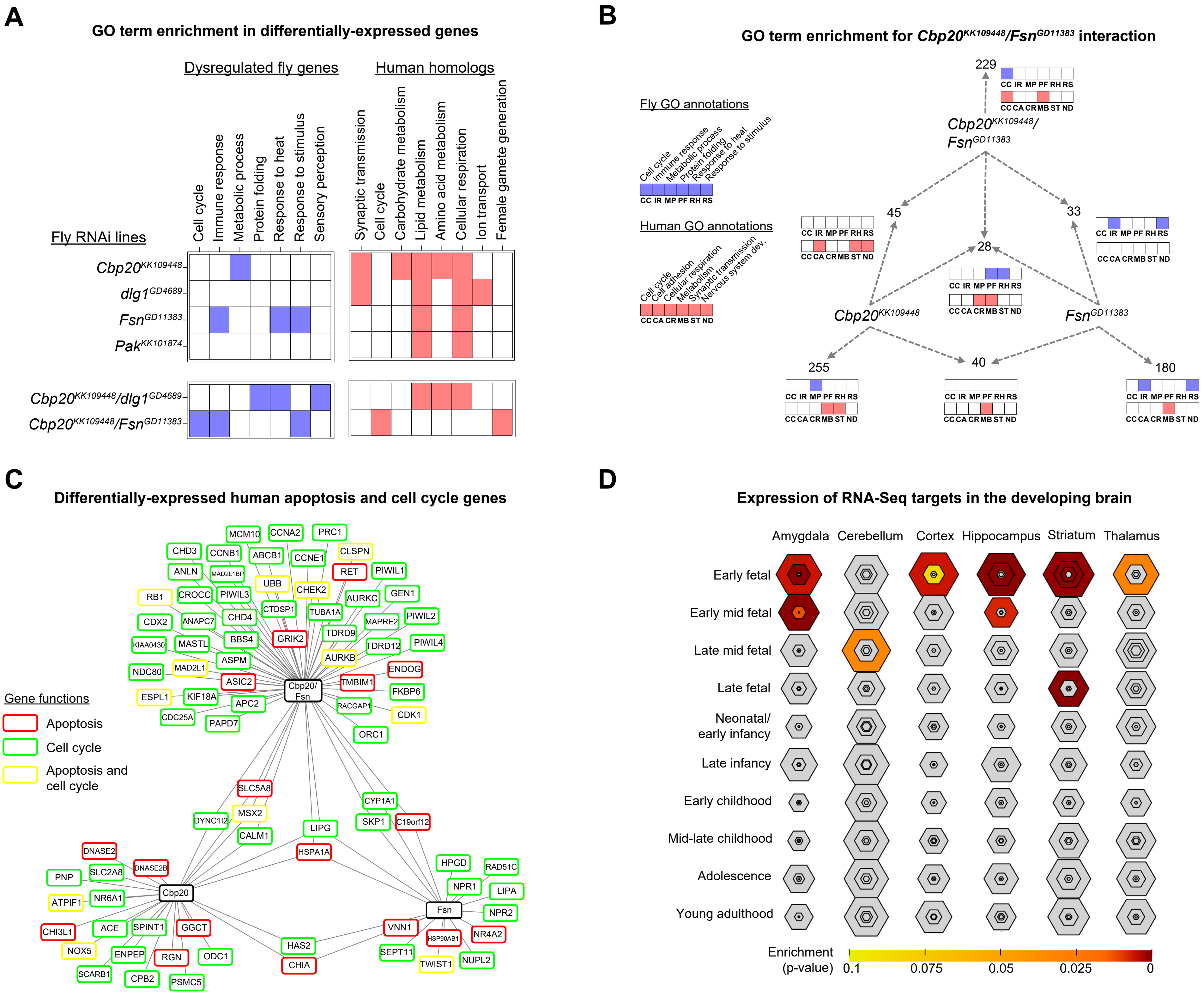

A
GO term enrichment in differentially-expressed genes
Dysregulated fly genes
Human homologs
Fly RNAi lines
Cell cycle
Immune response
Metabolic process
Protein folding
Response to heat
Response to stimulus
Sensory perception
Synaptic transmission
Cell cycle
Carbohydrate metabolism
Lipid metabolism
Amino acid metabolism
Cellular respiration
Ion transport
Female gamete generation
Cbp20KK109448
dlg1GD4689
FsnGD11383
PakKK101874
Cbp20KK109448/dlg1GD4689
Cbp20KK109448/FsnGD11383
B
GO term enrichment for Cbp20KK109448/FsnGD11383 interaction
Fly GO annotations
Cell cycle
Immune response
Metabolic process
Protein folding
Response to heat
Response to stimulus
CC IR MP PF RH RS
Human GO annotations
Cell cycle
Cell adhesion
Cellular respiration
Metabolism
Synaptic transmission
Nervous system dev.
CC CA CR MB ST ND
229
Cbp20KK109448/
FsnGD11383
45
33
28
Cbp20KK109448
FsnGD11383
255
40
180
C
Differentially-expressed human apoptosis and cell cycle genes
Gene functions
Apoptosis
Cell cycle
Apoptosis and cell cycle
Cbp20/Fsn
Cbp20
Fsn
MCM10
CCNA2
PRC1
CHD3
CCNB1
ABCB1
CCNE1
CLSPN
ANLN
MAD2L1BP
UBB
RET
PIWIL1
RB1
CROCC
PIWIL3
CHEK2
GEN1
CDX2
ANAPC7
CHD4
CTDSP1
TUBA1A
AURKC
PIWIL2
KIAA0430
MASTL
BBS4
GRIK2
TDRD9
MAPRE2
PIWIL4
NDC80
MAD2L1
ASPM
AURKB
TDRD12
ENDOG
ESPL1
KIF18A
ASIC2
TMBIM1
FKBP6
CDC25A
APC2
RACGAP1
CDK1
PAPD7
ORC1
SLC5A8
CYP1A1
DYNC1I2
MSX2
SKP1
C19orf12
CALM1
LIPG
HSPA1A
DNASE2
DNASE2B
PNP
SLC2A8
HPGD
RAD51C
NPR1
ATPIF1
NR6A1
LIPA
NPR2
CHI3L1
ACE
SPINT1
GGCT
VNN1
NR4A2
HSP90AB1
HAS2
NOX5
ENPEP
RGN
ODC1
SEPT11
NUPL2
CHIA
TWIST1
SCARB1
CPB2
PSMC5
D
Expression of RNA-Seq targets in the developing brain
Amygdala
Cerebellum
Cortex
Hippocampus
Striatum
Thalamus
Early fetal
Early mid fetal
Late mid fetal
Late fetal
Neonatal/ early infancy
Late infancy
Early childhood
Mid-late childhood
Adolescence
Young adulthood
Enrichment (p-value)
0.1
0.075
0.05
0.025
0
