# Supplementary material for: NCBP2 modulates neurodevelopmental defects of the 3q29 deletion in Drosophila and Xenopus laevis models
Source: PLoS Genet. 2020 Feb 13;16(2):e1008590. doi: 10.1371/journal.pgen.1008590 (PMC7043793; doi:10.1371/journal.pgen.1008590)
Supplement: S10 Fig — (A) Cellular phenotypes of flies with overexpression of Diap1 and Dronc. Representative brightfield adult eye images (scale bar = 100 μm) and confocal images of larval eye discs (scale bar = 30 μm) stained with anti-dcp1 are shown for flies with GMR-GAL4 overexpression of Diap1 and Dronc, while confocal images of pupal eyes (scale bar = 5 μm) stained with anti-DLG are also shown for flies with overexpression of Diap1. While the overexpression of Diap1 did not lead to any changes in the pupal or adult eye phenotype, overexpression of Dronc resulted in a large increase in apoptosis and depigmentation in the adult eye. (B) Box plot of Flynotyper distance ommatidial disorderliness (OD) scores for flies with concomitant GMR-GAL4 RNAi knockdown of Cbp20 or dlg1 and overexpression of Diap1 or Dronc is shown (n = 8–9, *p < 0.05, two-tailed Mann–Whitney test with Benjamini-Hochberg correction). (C) Box plot of Flynotyper angle OD scores for flies with knockdown of Cbp20 or dlg1 and overexpression of Diap1 or Dronc is shown (n = 8–9, *p < 0.05, two-tailed Mann–Whitney test with Benjamini-Hochberg correction). The distance and angle OD scores, component subscores derived from Flynotyper [53], mirror the trends observed in the overall phenotypic scores (Fig 6B). (D) Box plot of adult eye area in flies with knockdown of Cbp20 or dlg1 and overexpression of Diap1 or Dronc is shown (n = 8–9, *p < 0.05, two-tailed Mann–Whitney test with Benjamini-Hochberg correction). (E) Confocal images of pupal eyes (scale bar = 5 μm) stained with Phalloidin illustrate the rescue of photoreceptor cell organization defects due to knockdown of Cbp20 or dlg1 upon overexpression of Diap1. (F) Larval eye discs (scale bar = 30 μm) stained with TUNEL show rescue of apoptosis phenotypes observed in flies with knockdown of Cbp20 or dlg1 and overexpression of Diap1, as well as enhanced apoptosis with overexpression of Dronc. (G) Box plot of TUNEL-positive cells in the larval eye discs of flies with knockdow [file pgen.1008590.s010.pdf]

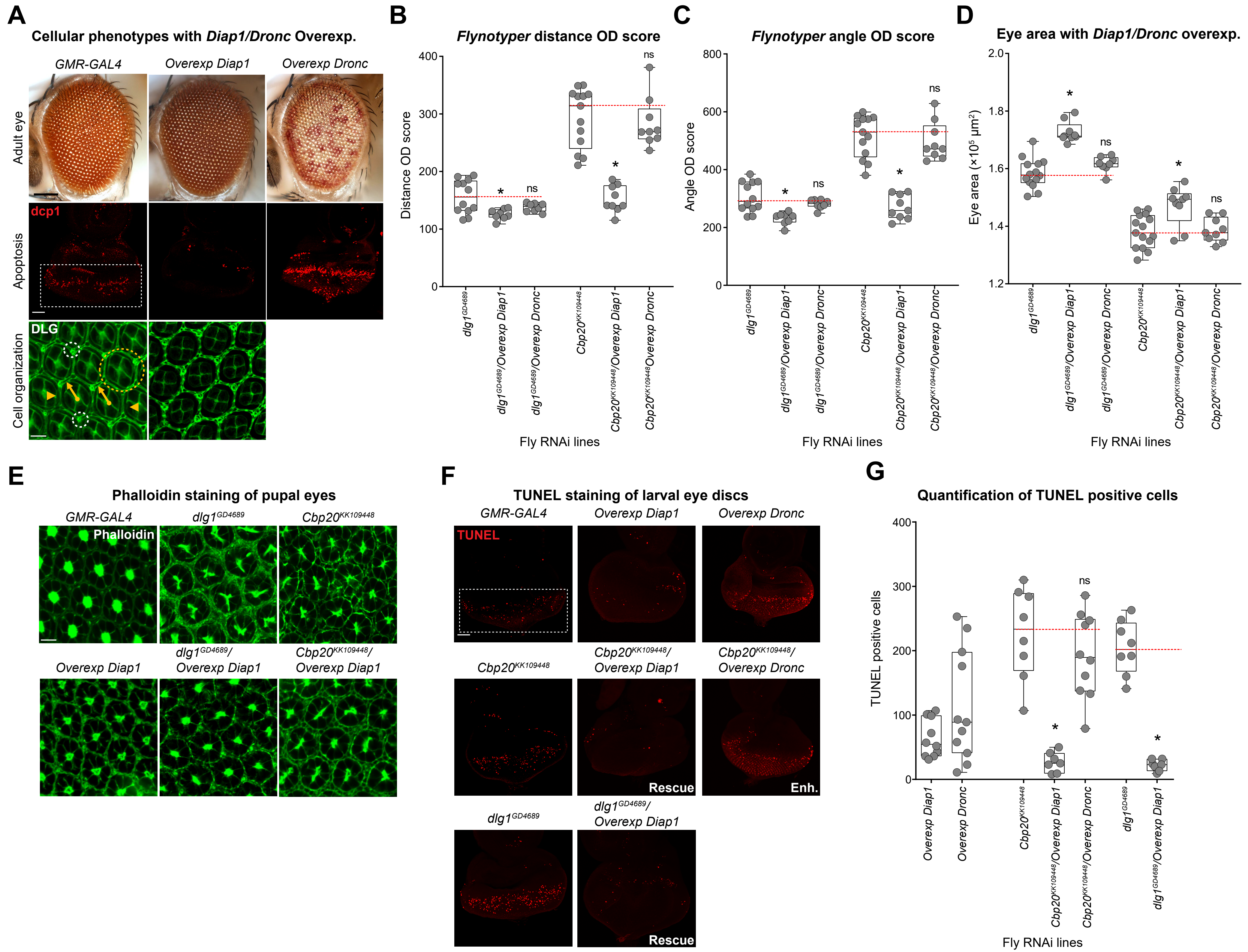
A
Cellular phenotypes with Diap1/Dronc Overexp.
GMR-GAL4
Overexp Diap1
Overexp Dronc
Adult eye
Apoptosis
dcp1
Cell organization
DLG
B
Flynotyper distance OD score
Distance OD score
Fly RNAi lines
C
Flynotyper angle OD score
Angle OD score
Fly RNAi lines
D
Eye area with Diap1/Dronc overexp.
Eye area (×10⁵ μm²)
Fly RNAi lines
dlg1 GD4689
dlg1 GD4689/Overexp Diap1
dlg1 GD4689/Overexp Dronc
Cbp20 KK109448
Cbp20 KK109448/Overexp Diap1
Cbp20 KK109448/Overexp Dronc
E
Phalloidin staining of pupal eyes
Phalloidin
GMR-GAL4
dlg1 GD4689
Cbp20 KK109448
Overexp Diap1
dlg1 GD4689/ Overexp Diap1
Cbp20 KK109448/ Overexp Diap1
F
TUNEL staining of larval eye discs
TUNEL
GMR-GAL4
Overexp Diap1
Overexp Dronc
Cbp20 KK109448
Cbp20 KK109448/ Overexp Diap1
Cbp20 KK109448/ Overexp Dronc
Rescue
Enh.
dlg1 GD4689
dlg1 GD4689/ Overexp Diap1
Rescue
G
Quantification of TUNEL positive cells
TUNEL positive cells
Overexp Diap1
Overexp Dronc
Cbp20 KK109448
Cbp20 KK109448/Overexp Diap1
Cbp20 KK109448/Overexp Dronc
dlg1 GD4689
dlg1 GD4689/Overexp Diap1
Fly RNAi lines
