# Supplementary material for: NCBP2 modulates neurodevelopmental defects of the 3q29 deletion in Drosophila and Xenopus laevis models
Source: PLoS Genet. 2020 Feb 13;16(2):e1008590. doi: 10.1371/journal.pgen.1008590 (PMC7043793; doi:10.1371/journal.pgen.1008590)
Supplement: S12 Fig — (A) Electrophoretic gels show decreased expression of homologs of 3q29 genes due to morpholino (MO) knockdown at various concentrations in X. laevis embryos. Three replicates (uninjected and two MO concentrations) were performed for each morpholino, and band intensities were compared with expression of ODC1 controls taken from the same cDNA samples and run on gels processed in parallel. (B) Quantification of expression for homologs of 3q29 genes at different MO concentrations, as measured by band intensity ratio to ODC1 controls (n = 3 replicates, *p<0.05, two-tailed Welch’s T-test with Benjamini-Hochberg correction). (C) Full images of western blots for quantification of cleaved caspase-3 levels in X. laevis embryos with MO knockdown of homologs of 3q29 genes. Two replicate experiments were performed, and the intensity of bands at 19kD and 17kD (green arrows), corresponding with cleaved caspase-3, were normalized to those for the β-actin loading controls. Embryos injected with control MO, uninjected embryos, and embryos treated with 30% EtOH as a positive control were included with the embryos injected with 3q29 MOs. (PDF) [file pgen.1008590.s012.pdf]

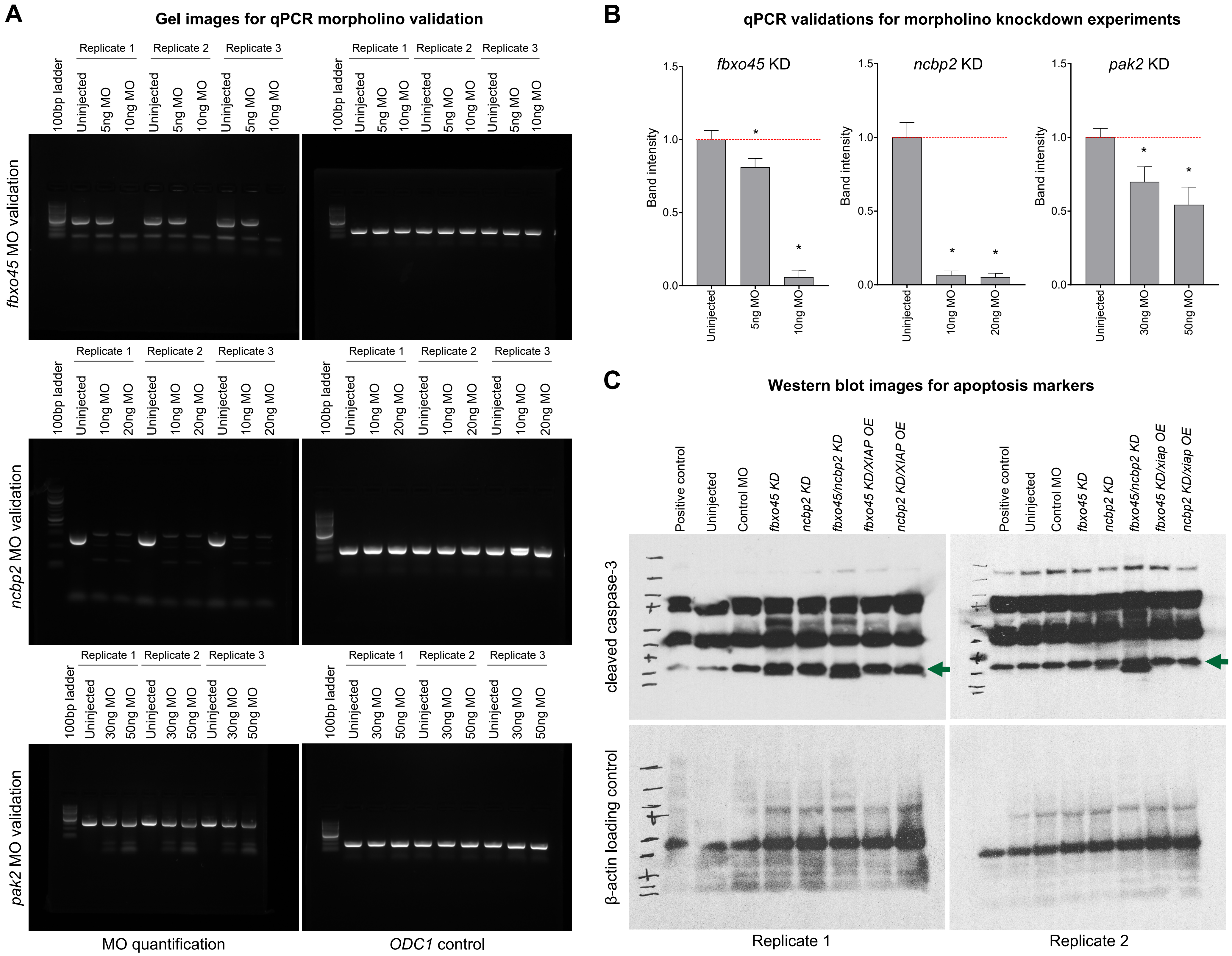

A
Gel images for qPCR morpholino validation
Replicate 1
Replicate 2
Replicate 3
100bp ladder
Uninjected
5ng MO
10ng MO
fbxo45 MO validation
10ng MO
20ng MO
ncbp2 MO validation
30ng MO
50ng MO
pak2 MO validation
MO quantification
ODC1 control
B
qPCR validations for morpholino knockdown experiments
fbxo45 KD
ncbp2 KD
pak2 KD
Band intensity
1.5
1.0
0.5
0.0
*
C
Western blot images for apoptosis markers
Positive control
Control MO
fbxo45 KD
ncbp2 KD
fbxo45/ncbp2 KD
fbxo45 KD/XIAP OE
ncbp2 KD/XIAP OE
fbxo45 KD/xiap OE
ncbp2 KD/xiap OE
cleaved caspase-3
β-actin loading control
Replicate 1
Replicate 2
