# Supplementary material for: NCBP2 modulates neurodevelopmental defects of the 3q29 deletion in Drosophila and Xenopus laevis models
Source: PLoS Genet. 2020 Feb 13;16(2):e1008590. doi: 10.1371/journal.pgen.1008590 (PMC7043793; doi:10.1371/journal.pgen.1008590)
Supplement: S13 Fig — (A) Representative eye images of stage 42 X. laevis tadpoles with MO knockdown of homologs of 3q29 genes (scale bar = 500 μm) show defects in eye size and morphology compared with the control (top). These defects were rescued with co-injection and overexpression of mRNA for homologs of 3q29 genes, as well as overexpression of the apoptosis inhibitor xiap for ncbp2 (bottom). (B) Box plot of eye area in X. laevis models with knockdown of homologs of 3q29 genes, normalized to controls, is shown (n = 48–71, *p < 0.05, two-tailed Welch’s T-test with Benjamini-Hochberg correction). Models with ncbp2 knockdown and xiap overexpression showed an increased eye size compared with ncbp2 knockdown. (C) Box plot of eye area in X. laevis models with knockdown of homologs of 3q29 genes and overexpression of mRNA for homologs of 3q29 genes, normalized to controls, is shown (n = 56–63, *p < 0.05, two-tailed Welch’s T-test with Benjamini-Hochberg correction). All boxplots indicate median (center line), 25th and 75th percentiles (bounds of box), and minimum and maximum (whiskers), with red dotted lines representing the control median. The data shown for the eye area experiments represent pooled results of three experimental batches, and were normalized to the respective controls from each batch. (PDF) [file pgen.1008590.s013.pdf]

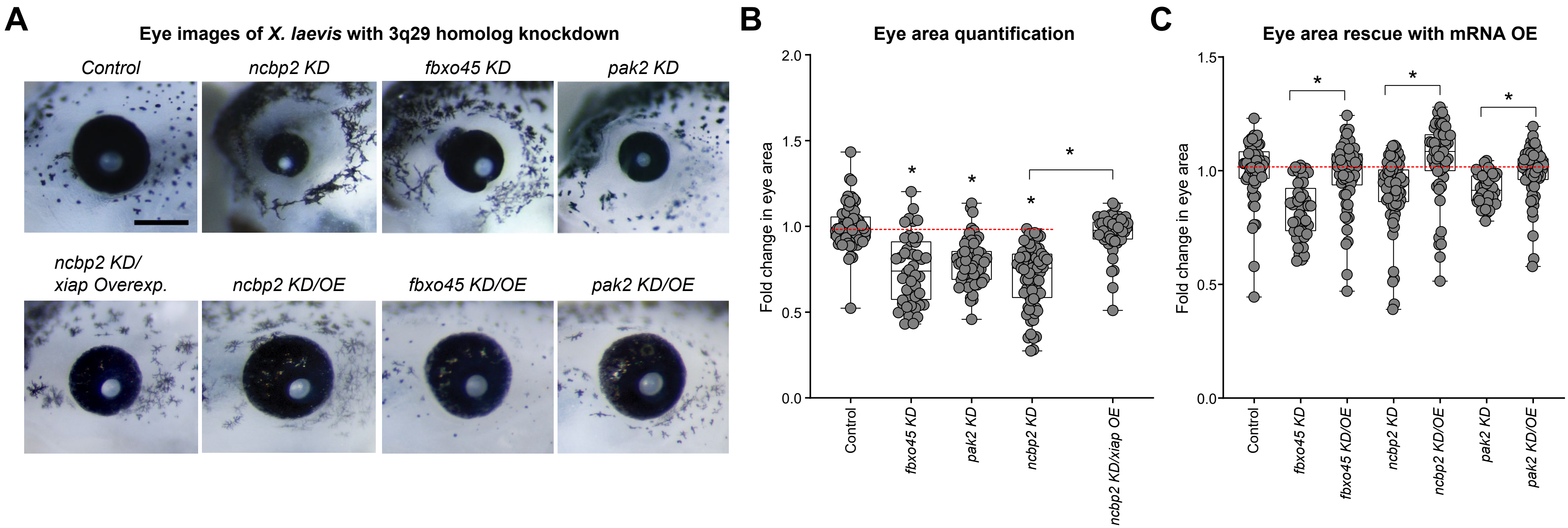

A
Eye images of X. laevis with 3q29 homolog knockdown
Control
ncbp2 KD
fbxo45 KD
pak2 KD
ncbp2 KD/
xiap Overexp.
ncbp2 KD/OE
fbxo45 KD/OE
pak2 KD/OE
B
Eye area quantification
Fold change in eye area
2.0
1.5
1.0
0.5
0.0
*
Control
fbxo45 KD
pak2 KD
ncbp2 KD
ncbp2 KD/xiap OE
C
Eye area rescue with mRNA OE
Fold change in eye area
1.5
1.0
0.5
0.0
*
Control
fbxo45 KD
fbxo45 KD/OE
ncbp2 KD
ncbp2 KD/OE
pak2 KD
pak2 KD/OE
