# Supplementary material for: NCBP2 modulates neurodevelopmental defects of the 3q29 deletion in Drosophila and Xenopus laevis models
Source: PLoS Genet. 2020 Feb 13;16(2):e1008590. doi: 10.1371/journal.pgen.1008590 (PMC7043793; doi:10.1371/journal.pgen.1008590)
Supplement: S2 Table — Elav-GAL4 flies were crossed with RNAi lines of fly homologs of 3q29 genes at 25°C, and 3–4 day old adult Drosophila heads were used to quantify the level of expression compared with Elav-GAL4 controls. Elav-GAL4;;UAS-Dicer2 flies crossed with CG5359 flies showed overexpression of tiptop [103] and were therefore excluded from further experiments. A list of full genotypes for fly crosses used in these experiments is provided in S2 File, and statistics for these data are provided in S5 File. (PDF) [file pgen.1008590.s016.pdf]

Gene name	Forward and reverse primers	RNAi knockdown (% expression)
app^{KK108227}	For-5'- GCGATCAGACAACCAACGAG-3'<br>Rev-5'- CGCCTTTGGAGGAGAAGGAT-3'	55.457
Cbp20^{KK109448}	For-5'- TTGTGAATGGCACTCGCTTG-3'<br>Rev-5'- GTCCCAGTCCACACGAATCA-3'	43.900
CG5359^{KK107839}	For-5'- ACGTTATGGCCGAGAAACTCA-3'<br>Rev-5'-TGGCGACGTCCTTGTCATAG-3'	20.945
CG5543^{KK109031}	For-5'- AAATCCACTTAGCGTGGGGC-3'<br>Rev-5'- AGGAAATTTTACCGCGTTGCAT-3'	49.764
CG6836^{KK112485}	For-5'- CCCTTCATCGTCTGCTCCAT-3'<br>Rev-5'- GTGATTTGGAGGGACCAAGC-3'	49.087
CG8888^{GD3777}	For-5'- TTCGCAAGAGCTTGGACCTC-3'<br>Rev-5'- TTTGTGTTAGCCGAGCGGAA-3'	25.005
CG8892^{GD14061}	For-5'- TCCAGAGCAACGTCATGTCC-3'<br>Rev-5'- TGGACCGTCTGTTAAGTGCC-3'	38.721
dlg1^{GD4689}	For-5'- ACACAAGACGATGCCAATGC-3'<br>Rev-5'- TCCACCCTGTAGATAATCTCGC-3'	62.691
Fsn^{GD11383}	For-5'- CCCATTTGGTTGGTGTGGGA-3'<br>Rev-5'- TGGATTTACCCGTTCCTGTTGA-3'	55.230
Pak^{KK101874}	For-5'- GTCGTCACCCGGAAACAGTA-3'<br>Rev-5'- GCCCAAAGACCAAAGGTCCA-3'	40.212
Pcyt2^{KK110819}	For-5'- CGCTACGTGGATGAGATCGT-3'<br>Rev-5'- TCCTCATTTAGCGTCCACGG-3'	80.642
PIG-X^{KK109717}	For-5'- TGACCTGCAGCGTTTGAAGA-3'<br>Rev-5'- TGACGAACTTAGGATAGATGGCA-3'	29.775
PIG-Z^{KK107404}	For-5'-TCCAGAGCGTGGAGGTAATG-3'<br>Rev-5'- CGTATGCTCCAGCCGAAAGT-3'	37.856
Ulp1^{GD7581}	For-5'-CCTGGCCAAGGGCTAAAAGT-3'<br>Rev-5'- GACATGCGTGTGTTCGCTAC-3'	30.077
Rp49 control	For-5'-GCAAGCCCAAGGGTATCGA-3'<br>Rev-5'-ACCGATGTTGGGCATCAGA-3'	--
tiptop	For-5'-CCTCCACAGCATCAGCAACA-3'<br>Rev-5'-CCACCAGGTCGTTACCGTTC-3'	--
