# Supplementary material for: NCBP2 modulates neurodevelopmental defects of the 3q29 deletion in Drosophila and Xenopus laevis models
Source: PLoS Genet. 2020 Feb 13;16(2):e1008590. doi: 10.1371/journal.pgen.1008590 (PMC7043793; doi:10.1371/journal.pgen.1008590)
Supplement: S4 Table — Individual larval eye disc images were assigned mild, moderate or severe scores based on the severity of axon projection loss observed in each eye disc (see Methods). We found that the mild to moderate defects observed with knockdown of Cbp20 were enhanced with concomitant knockdown of dlg1 or Fsn, while Diap1 overexpression partially rescued the defects observed with knockdown of Cbp20 or dlg1. A list of full genotypes for fly crosses used in these experiments is provided in S2 File. (PDF) [file pgen.1008590.s018.pdf]

**RNAi line**	**Mild axon targeting phenotypes**	**Moderate axon targeting phenotypes**	**Severe axon targeting phenotypes**
Cbp20^{KK109448}	4/9	3/9	2/9
dlg1^{GD4689}	0/7	2/7	5/7
Fsn^{GD11383}	7/20	7/20	6/20
Pak^{KK101874}	2/8	4/8	2/8
Cbp20^{KK109448}/ *dlg1*^{GD4689}	2/17	8/17	7/17
Cbp20^{KK109448}/ *Fsn*^{GD11383}	1/16	4/16	11/16
Cbp20^{KK109448}/ *Overexp. Diap1*	5/11	6/11	0/11
dlg1^{GD4689}/ *Overexp. Diap1*	1/17	8/17	8/17
