# Supplementary material for: NCBP2 modulates neurodevelopmental defects of the 3q29 deletion in Drosophila and Xenopus laevis models
Source: PLoS Genet. 2020 Feb 13;16(2):e1008590. doi: 10.1371/journal.pgen.1008590 (PMC7043793; doi:10.1371/journal.pgen.1008590)
Supplement: S5 Table — Table comparing Flynotyper scores for flies with GMR-GAL4;UAS-Dicer2 RNAi knockdown of homologs of 3q29 genes (shaded in grey) with previously published scores for flies with GMR-GAL4;UAS-Dicer2 RNAi knockdown of homologs of candidate neurodevelopmental genes [53]. (PDF) [file pgen.1008590.s019.pdf]

Fly RNAi line	Human homolog	CNV region	Avg. eye phenotypic score
Ube3a^{KK104898}	*UBE3A*	Core gene	59.733
Pten^{GD13500}	*PTEN*	Core gene	58.275
Cadps$^{GD9502\_1}$	*CADPS2*	Core gene	56.758
PIG-Z^{KK107404}	*PIGZ*	3q29	56.243
arm^{KK102545}	*CTNNB1*	Core gene	54.865
app^{KK108227}	*ZDHHC19*	3q29	53.614
kis^{GD16331}	*CHD8*	Core gene	51.182
Nrx-1^{GD2619}	*NRXN1*	Core gene	48.753
Prosap^{GD10101}	*SHANK3*	Core gene	48.748
Cbp20^{KK109448}	*NCBP2*	3q29	46.268
dlg1^{GD4689}	*DLG1*	3q29	43.219
CG5543^{KK109031}	*WDR53*	3q29	40.349
CG8888^{GD3777}	*BDH1*	3q29	39.126
rk$^{GD14383\_1}$	*LGR5*	Core gene	38.021
MCPH1$^{GD12537\_2}$	*MCPH1*	Core gene	36.835
Pak^{KK101874}	*PAK2*	3q29	36.691
para$^{GD3392\_1}$	*SCN1A*	Core gene	35.846
PIG-X^{KK109717}	*PIGX*	3q29	34.392
Eph^{GD39}	*EPHA6*	Core gene	31.468
CG8892^{GD14061}	*UBXN7*	3q29	31.179
CG6836^{KK112485}	*OSTalpha*	3q29	30.842
Ulp1^{GD7581}	*SENP5*	3q29	30.383
Pcyt2^{KK110819}	*PCYT1A*	3q29	28.423
Fsn^{GD11383}	*FBXO45*	3q29	27.671
