# Supplementary material for: NCBP2 modulates neurodevelopmental defects of the 3q29 deletion in Drosophila and Xenopus laevis models
Source: PLoS Genet. 2020 Feb 13;16(2):e1008590. doi: 10.1371/journal.pgen.1008590 (PMC7043793; doi:10.1371/journal.pgen.1008590)
Supplement: S6 Table — The number of “+” symbols displayed in the table indicate the severity of the observed cellular defects. Note that n = 4–16 pupal eye preparations were assessed for each RNAi line tested. A list of full genotypes for fly crosses used in these experiments is provided in S2 File. (PDF) [file pgen.1008590.s020.pdf]

RNAi line	Cone cell defect	Primary cell defect	Secondary cell defect	Bristle group defect	Rotation error	Hexagonal defect	Photoreceptor defect
GMR-GAL4							
Cbp20^{KK109448}	++	+	++	++	++	++	+++
CG5543^{KK109031}	++	++	++	+++	++	+	+
CG6836^{KK112485}	+			+	+		+
CG8888^{GD3777}	++			+	++		++
CG8892^{GD14061}							+
dlg1^{GD4689}	++		+	+++	+	++	+++
Fsn^{GD11383}	++	+		++	++	+	
Pak^{KK101874}	+			+	+	+	
Pcyt2^{KK110819}	+	++	++	++	++	+	+
PIG-X^{KK109717}	+		+	++	++		+
PIG-Z^{KK107404}	+		+	++	+	+	++
Ulp1^{GD7581}	+	++	++	+	++	+	+
