# Supplementary material for: NCBP2 modulates neurodevelopmental defects of the 3q29 deletion in Drosophila and Xenopus laevis models
Source: PLoS Genet. 2020 Feb 13;16(2):e1008590. doi: 10.1371/journal.pgen.1008590 (PMC7043793; doi:10.1371/journal.pgen.1008590)
Supplement: S7 Table — “All interactions” indicates the number of pairwise crosses where at least one second-hit RNAi or mutant line showed enhancement of the single-hit phenotype, while “Validated” indicates the number of interactions which have two or more crosses with a second-hit RNAi or mutant line (if available) showing the same result. “Reciprocal cross” indicates the number of interactions with concordant results across pairs of reciprocal cross (i.e. Cbp20/dlg1 vs. dlg1/Cbp20). These totals include crosses with the mutant line Tsf2KG01571, as eye-specific RNAi knockdown of Tsf2 was lethal, as well as flies heterozygous for dlg1 RNAi and homozygous for Cbp20 RNAi. Crosses with other RNAi or mutant lines for the same homolog (shaded in grey) are included as validation lines tested but were not counted as interactions. A list of full genotypes for fly crosses used in these experiments is provided in S2 File. (PDF) [file pgen.1008590.s021.pdf]

Second-hit homolog	*app*^{KK108227}	*Cbp20*^{KK109448}	*CG6836*^{KK112485}	*CG8888*^{GD3777}	*dlg1*^{GD4689}	*Fsn*^{GD11383}	*Pak*^{KK101874}	*PIG-X*^{KK109717}	*PIG-Z*^{KK107404}
app	NA	Enhancer (1/1)	No interaction (0/1)	No interaction (0/1)	Enhancer (1/1)	NA	No interaction (0/1)	No interaction (0/1)	No interaction (0/1)
Cbp20	Enhancer (1/1)	Enhancer (3/3)	Enhancer (1/1)	Enhancer (3/3)	Enhancer (2/2)	Enhancer (2/3)	Enhancer (3/3)	No interaction (0/1)	Enhancer (3/3)
CG6836	No interaction (0/1)	Enhancer (1/1)	NA	Enhancer (1/1)	Enhancer (1/1)	No interaction (0/1)	No interaction (0/1)	No interaction (0/1)	No interaction (0/1)
CG8888	No interaction (0/1)	Enhancer (3/3)	Enhancer (1/1)	Not validated (1/2)	Not validated (1/3)	Enhancer (2/3)	No interaction (0/3)	No interaction (0/1)	Enhancer (3/3)
dlg1	Enhancer (1/1)	Enhancer (4/4)	No interaction (0/1)	Not validated (1/2)	Enhancer (1/1)	Not validated (1/2)	Not validated (1/2)	No interaction (0/1)	Enhancer (3/3)
Fsn	Enhancer (1/1)	Enhancer (3/3)	No interaction (0/1)	Not validated (1/3)	Not validated (1/3)	No interaction (0/2)	No interaction (0/2)	No interaction (0/1)	Enhancer (2/3)
Pak	No interaction (0/1)	Enhancer (3/3)	No interaction (0/1)	Not validated (1/3)	Not validated (1/3)	No interaction (0/1)	No interaction (0/1)	No interaction (0/1)	Enhancer (2/3)
PIG-X	No interaction (0/1)	Enhancer (1/1)	No interaction (0/1)	No interaction (0/1)	No interaction (0/1)	No interaction (0/1)	No interaction (0/1)	NA	No interaction (0/1)
PIG-Z	No interaction (0/1)	Enhancer (2/2)	No interaction (0/1)	Enhancer (2/2)	Not validated (1/2)	Not validated (1/2)	No interaction (0/2)	No interaction (0/1)	Enhancer (1/1)
CG5543	NA	Enhancer (2/2)	NA	Enhancer (2/2)	Not validated (1/2)	NA	Not validated (1/2)	NA	NA
CG8892	NA	Enhancer (1/1)	NA	No interaction (0/1)	Enhancer (1/1)	NA	No interaction (0/1)	NA	NA
Pcyt2	NA	Enhancer (1/1)	NA	Enhancer (1/1)	No interaction (0/1)	NA	Enhancer (1/1)	NA	Enhancer (1/1)
Tsf2	NA	Enhancer (1/1)	NA	Enhancer (1/1)	No interaction (0/1)	No interaction (0/1)	Enhancer (1/1)	NA	Enhancer (1/1)
Ulp1	NA	No interaction (0/2)	NA	Not validated (1/2)	Not validated (1/2)	NA	Not validated (1/2)	NA	NA
Lines tested **(161 total)**	8	28	8	25	24	16	23	8	21
All interactions **(54/94 total)**	3/8	12/13	2/8	10/13	10/13	4/8	6/13	0/8	7/10
Validated **(39/94 total)**	3/8	12/13	2/8	6/13	4/13	2/8	3/13	0/8	7/10
Reciprocal cross **(19/26 total)**	2/2	7/8	2/2	3/3	1/3	1/2	1/1	0/0	2/5
