# Supplementary material for: NCBP2 modulates neurodevelopmental defects of the 3q29 deletion in Drosophila and Xenopus laevis models
Source: PLoS Genet. 2020 Feb 13;16(2):e1008590. doi: 10.1371/journal.pgen.1008590 (PMC7043793; doi:10.1371/journal.pgen.1008590)
Supplement: S8 Table — The number of “+” symbols displayed in the table indicate the severity of the observed cellular defects. Note that n = 4–16 pupal eye preparations were assessed for each interaction cross tested. A list of full genotypes for fly crosses used in these experiments is provided in S2 File. (PDF) [file pgen.1008590.s022.pdf]

Pairwise cross	Cone cell defects			Primary cell defect	Secondary cell defect	Bristle cell defect	Rotation error	Hexagonal defect	Photoreceptor defect
	Number error	Arrangement error	Orientation error						
dlg1^{GD4689}			++		+	+++	+	++	+++
Cbp20^{KK109448}			++	+	++	++	++	++	+++
Cbp20^{KK109448}/ *CG8888*^{GD3777}	+	++	++	++	++	++	++	+++	++++
Cbp20^{KK109448}/ *dlg1*^{GD4689}	+	++	++	++	++	+++	++	+	++++
Cbp20^{KK109448}/ *Fsn*^{GD11383}	+	++	++	++	+++	+++	++	++++	++++
Cbp20^{KK109448}/ *Pak*^{KK101874}		++	++	+	++	++	+	+	+++
Cbp20^{KK109448}/ *PIG-Z*^{KK107404}			+	++	++	+++	++	+++	++++
Overexp Diap1									
Cbp20^{KK109448}/ *Overexp Diap1*			+		++	+			
dlg1^{GD4689}/ *Overexp Diap1*			++			+++			
