# Supplementary material for: NCBP2 modulates neurodevelopmental defects of the 3q29 deletion in Drosophila and Xenopus laevis models
Source: PLoS Genet. 2020 Feb 13;16(2):e1008590. doi: 10.1371/journal.pgen.1008590 (PMC7043793; doi:10.1371/journal.pgen.1008590)
Supplement: S9 Table — “All interactions” indicates the number of crosses where at least one second-hit RNAi line showed enhancement of the single-hit phenotype, while “Validated interactions” indicates the number of interactions which have two or more crosses with a second-hit RNAi or mutant line (if available) showing the same result. Results from two distinct fly homologs of CHRNA7 that were crossed with homologs of 3q29 genes, nAChRα6 and nAChRα7, were combined for the final number of interactions. Shaded interactions indicate pairwise crosses where the phenotypes observed with knockdown of the homolog for the neurodevelopmental gene by itself were suppressed with concomitant knockdown of homologs for 3q29 genes. The neurodevelopmental genes are annotated for cell cycle/apoptosis function (Gene Ontology terms GO:0007049 and GO:0006915) and association with microcephaly disorders [65]. A list of full genotypes for fly crosses used in these experiments is provided in S2 File. (PDF) [file pgen.1008590.s023.pdf]

Second-hit homolog	Cell cycle/ apoptosis	Microcephaly	***Cbp20***^{KK109448}	***CG8888***^{GD3777}	***dlg1***^{GD4689}	***Pak***^{KK101874}
Arm (CTNNB1)	X		Enhancer (2/2)	Enhancer (2/2)	Enhancer (2/2)	Enhancer (2/2)
Asp (ASPM)	X	X	Enhancer (1/1)	Enhancer (1/1)	No interaction (0/1)	No interaction (0/1)
Cadps (CADPS2)			Enhancer (2/2)	Not validated (1/2)	Not validated (1/2)	Enhancer (2/2)
Eph (EPHA6)			Enhancer (3/3)	Enhancer (3/3)	Enhancer (3/3)	Not validated (1/3)
kis (CHD8)	X		Not validated (1/2)	No interaction (0/2)	No interaction (0/2)	No interaction (0/2)
Klp61F (KIF11)	X	X	Enhancer (2/2)	Enhancer (1/1)	Enhancer (1/1)	Enhancer (1/1)
MCPH1 (MCPH1)	X	X	Enhancer (2/3)	Enhancer (3/3)	No interaction (0/3)	Not validated (1/3)
nAChRα6 nAChRα7 (CHRNA7)			Enhancer (3/5)	Enhancer (3/5)	Enhancer (3/5)	Enhancer (2/5)
Nrx-1 (NRXN1)			Enhancer (3/3)	Enhancer (3/3)	Enhancer (3/3)	No interaction (0/3)
para (SCN1A)			Enhancer (3/3)	Enhancer (2/3)	No interaction (0/3)	Not validated (1/3)
Prosap (SHANK3)			Not validated (1/2)	No interaction (0/2)	Not validated (1/2)	No interaction (0/2)
Pten (PTEN)	X		Enhancer (2/2)	Enhancer (2/2)	Enhancer (2/2)	Not validated (1/2)
Rk (LGR5)	X		Enhancer (4/5)	Enhancer (3/5)	No interaction (0/5)	No interaction (0/5)
Sas-4 (CENPJ)	X	X	Enhancer (2/2)	Enhancer (2/2)	Not validated (1/2)	No interaction (0/2)
Ube3a (UBE3A)			Enhancer (2/2)	Not validated (1/2)	No interaction (0/2)	Not validated (1/2)
**Lines tested (153)**			39	38	38	38
**All interactions (46/60)**			15/15	13/15	9/15	9/15
**Validated interactions (34/60)**			13/15	11/15	6/15	4/15
