# Supplementary material for: NCBP2 modulates neurodevelopmental defects of the 3q29 deletion in Drosophila and Xenopus laevis models
Source: PLoS Genet. 2020 Feb 13;16(2):e1008590. doi: 10.1371/journal.pgen.1008590 (PMC7043793; doi:10.1371/journal.pgen.1008590)
Supplement: S10 Table — Comparison of mice with heterozygous deletion of the syntenic 3q29 region [14,15] with heterozygous knockout mouse models for Dlg1 [14] and Pak2 [72]. Blue shaded boxes indicate phenotypes observed in the knockout models, while gray-shaded boxes indicate a phenotype that was not tested in the knockout model. Neither Dlg1+/- nor Pak2+/- knockout mice recapitulate the body and brain weight, spatial learning and memory, or acoustic startle defects observed in the deletion mouse models. (PDF) [file pgen.1008590.s024.pdf]

**3q29 deletion mouse models**	*B6J.Del16* $^{+/Bdh1\text{-}Tfrc}$ *(Baba et al.)*	*B6N.Del16* $^{+/Bdh1\text{-}Tfrc}$ *(Rutkowski et al.)*	*B6N.Dlg1* $^{+/-}$ *(Rutkowski et al.)*	*Pak2* $^{+/-}$ *(Wang et al.)*
Weight	Decreased	Decreased	No phenotype	Not tested
Brain size	Decreased	Decreased	Not tested	No phenotype
Locomotor activity	No phenotype	No phenotype	No phenotype	No phenotype
Amphetamine-induced locomotor activity	Not tested	Increased	Increased	Not tested
Anxiety (elevated plus maze or open field)	Not tested	No phenotype	No phenotype	No phenotype
Spatial learning and memory (water maze)	Not tested	Decreased	No phenotype	No phenotype
Acoustic startle response	Increased	Increased	No phenotype	No phenotype
Prepulse inhibition/sensorimotor gating	Decreased	No phenotype	No phenotype	No phenotype
Startle response w/risperidone	Rescued	Not tested	Not tested	Not tested
Marble burying	Not tested	No phenotype	No phenotype	Increased
Self-grooming	Increased	Not tested	Not tested	Increased
Social interaction (free or 3-chamber)	Decreased	Decreased	No phenotype	Decreased
Fear conditioning (context)	Decreased	No phenotype	No phenotype	Not tested
Auditory excitatory neuron activity	Increased	Not tested	Not tested	Not tested
Parvalbumin neuronal count	Decreased	Not tested	Not tested	Not tested
Dendritic spine density	Not tested	Not tested	Not tested	Decreased
Long-term potentiation	Not tested	Not tested	Not tested	Decreased
Synaptic density	Not tested	Not tested	Not tested	Decreased
Neuronal migration	Not tested	Not tested	Not tested	Decreased
Transcriptome	Immediate early signaling genes	Not tested	Not tested	Post-synaptic density, cytoskeleton, channel activity
