# Supplementary material for: NCBP2 modulates neurodevelopmental defects of the 3q29 deletion in Drosophila and Xenopus laevis models
Source: PLoS Genet. 2020 Feb 13;16(2):e1008590. doi: 10.1371/journal.pgen.1008590 (PMC7043793; doi:10.1371/journal.pgen.1008590)
Supplement: S11 Table — This table shows the number of candidate autism, intellectual disability and schizophrenia genes annotated for apoptosis function. The minimum, mean and maximum numbers of apoptosis genes in 100,000 simulated sets of candidate genes are shown, along with the percentiles and empirical p-values of the observed overlap with apoptosis genes for each simulation. (PDF) [file pgen.1008590.s025.pdf]

**Candidate gene set**	**Overlap with apoptosis (%)**	**Simulated overlap with apoptosis**			**Percentile of observed overlap**	**Empirical p-value**
		Min.	Mean	Max.		
Autism (n=756)	106 (14.0%)	40	71	104	100%	$p<1.00\times10^{-5}$
Intellectual disability (n=1,854)	265 (14.3%)	121	170	223	100%	$p<1.00\times10^{-5}$
Schizophrenia (n=2,546)	268 (10.5%)	180	237	302	98.6%	p=0.014
