# Supplementary material for: NCBP2 modulates neurodevelopmental defects of the 3q29 deletion in Drosophila and Xenopus laevis models
Source: PLoS Genet. 2020 Feb 13;16(2):e1008590. doi: 10.1371/journal.pgen.1008590 (PMC7043793; doi:10.1371/journal.pgen.1008590)
Supplement: S12 Table — (PDF) [file pgen.1008590.s026.pdf]

***X. laevis* homolog**	**Morpholino**
ncbp2	for L, 5’- CGGTTTCCCTAGAATAGAAACAGGT-3’
fbxo45	for L and S, 5’-TATCTGTGGTGGGAAGAAAAGGTCA-3’
dlg1	for L, 5’-CAAATGAGGCAGCAACTTACTTTCT-3’
pak2	for L and S, 5’-AGAGATAAATCCTACCTTTTTCTGT-3’
standard control	5′-cctcttacctcagttacaatttata-3′
