# Supplementary material for: NCBP2 modulates neurodevelopmental defects of the 3q29 deletion in Drosophila and Xenopus laevis models
Source: PLoS Genet. 2020 Feb 13;16(2):e1008590. doi: 10.1371/journal.pgen.1008590 (PMC7043793; doi:10.1371/journal.pgen.1008590)
Supplement: S13 Table — (PDF) [file pgen.1008590.s027.pdf]

***X. laevis* homolog**	**Primers**
ncbp2	forward for L allele 5’- ATCTGAGTCAGTATCGGGACC-3’<br>reverse for L allele 5’- CCCTTCCTTAAATCCTGCATCC-3’
fbxo45	forward for L and S allele 5’- CCGACATACTGTGCAACCTG-3’<br>reverse for L and S allele 5’-TGTCCAAGATCACCCGAATCC-3’
dlg1	forward for L allele 5’-CTCTCCTATGAACCCGTCAC-3’<br>reverse for L allele 5’-CCGGCCTCTATGAATTTGTG-3’
pak2	forward for L and S allele 5’-AGGATAAACCACCAGCTCCTC-3’<br>reverse for L and S allele 5’-GGGAGCCCATCTTTATCTGGTG-3’
ODC1 control	forward 5’- GCCATTGTGAAGACTCTCTCCATTC-3’<br>reverse 5’- TTCGGGTGATTCCTTGCCAC-3’
