# Supplementary material for: NCBP2 modulates neurodevelopmental defects of the 3q29 deletion in Drosophila and Xenopus laevis models
Source: PLoS Genet. 2020 Feb 13;16(2):e1008590. doi: 10.1371/journal.pgen.1008590 (PMC7043793; doi:10.1371/journal.pgen.1008590)
Supplement: S7 Fig — (A-F) Box plots of phenotypic scores for pairwise GMR-GAL4 RNAi knockdown of select fly homologs of 3q29 genes (Cbp20, CG8888, dlg1, Fsn, Pak, and PIG-Z) with validation RNAi and mutant lines for other homologs of 3q29 genes, compared with recombined lines for individual homologs of 3q29 genes crossed with controls, are shown (n = 4–14, *p < 0.05, two-tailed Mann–Whitney test with Benjamini-Hochberg correction), are shown. These crosses include flies homozygous for Cbp20 RNAi as well as flies homozygous for Cbp20 RNAi and heterozygous for dlg1 RNAi (green arrows). Note that the phenotypic scores derived from Flynotyper may not accurately capture the necrotic patches observed in these crosses. All boxplots indicate median (center line), 25th and 75th percentiles (bounds of box), and minimum and maximum (whiskers), with red dotted lines representing the control median. A list of full genotypes for fly crosses used in these experiments is provided in S2 File. (PDF) [file pgen.1008590.s007.pdf]

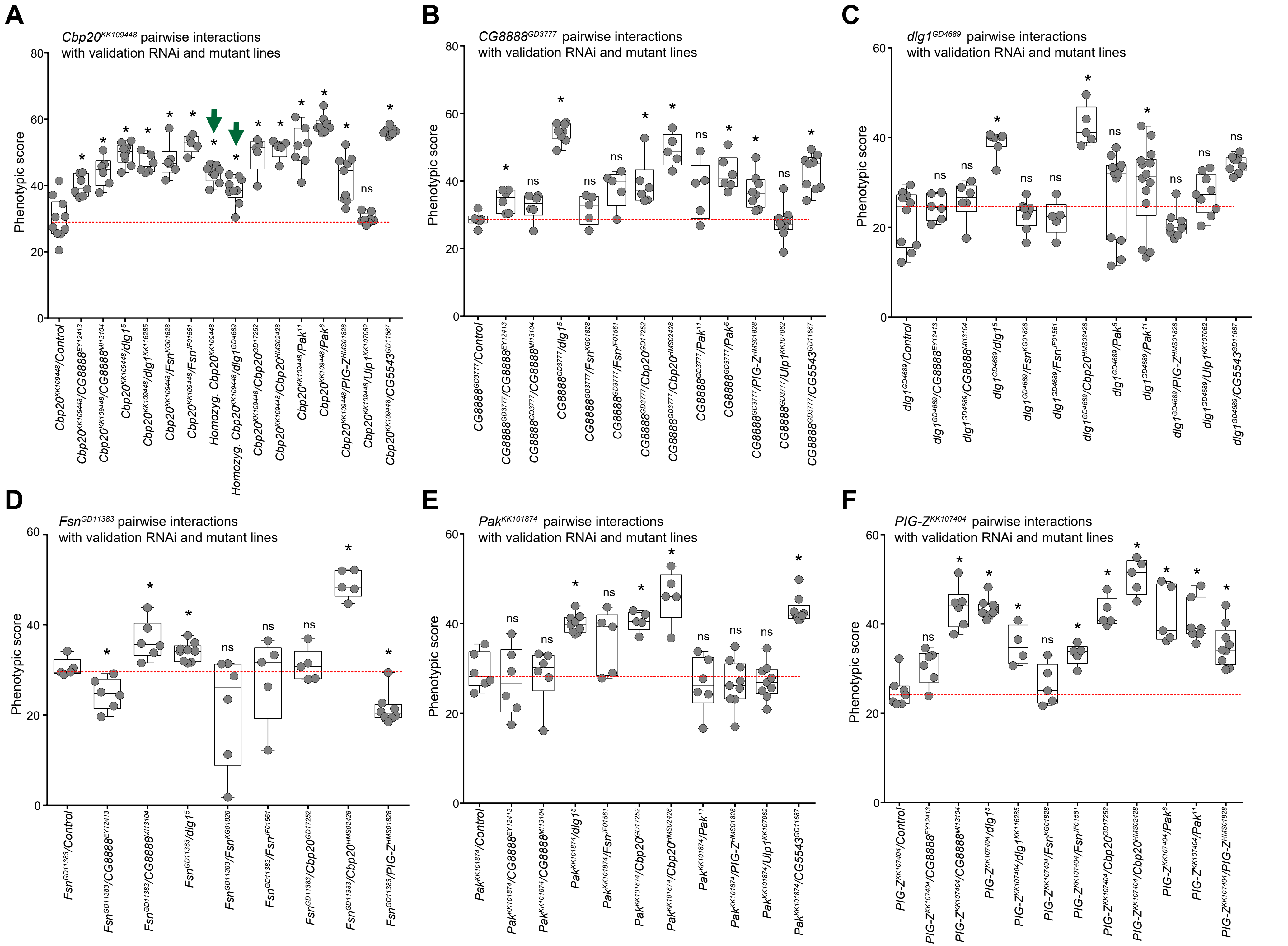
A
Cbp20KK109448 pairwise interactions
with validation RNAi and mutant lines
Phenotypic score
0
20
40
60
80
Cbp20KK109448/Control
Cbp20KK109448/CG8888EY12413
Cbp20KK109448/CG8888MI13104
Cbp20KK109448/dlg15
Cbp20KK109448/dlg1KK116285
Cbp20KK109448/FsnKG01828
Cbp20KK109448/FsnJF01561
Homozyg. Cbp20KK109448
Homozyg. Cbp20KK109448/dlg1GD4689
Cbp20KK109448/Cbp20GD17252
Cbp20KK109448/Cbp20HMS02428
Cbp20KK109448/Pak11
Cbp20KK109448/Pak6
Cbp20KK109448/PIG-ZHMS01828
Cbp20KK109448/Ulp1KK107062
Cbp20KK109448/CG5543GD11687
B
CG8888GD3777 pairwise interactions
with validation RNAi and mutant lines
Phenotypic score
0
20
40
60
80
CG8888GD3777/Control
CG8888GD3777/CG8888EY12413
CG8888GD3777/CG8888MI13104
CG8888GD3777/dlg15
CG8888GD3777/FsnKG01828
CG8888GD3777/FsnJF01561
CG8888GD3777/Cbp20GD17252
CG8888GD3777/Cbp20HMS02428
CG8888GD3777/Pak11
CG8888GD3777/Pak6
CG8888GD3777/PIG-ZHMS01828
CG8888GD3777/Ulp1KK107062
CG8888GD3777/CG5543GD11687
C
dlg1GD4689 pairwise interactions
with validation RNAi and mutant lines
Phenotypic score
0
20
40
60
dlg1GD4689/Control
dlg1GD4689/CG8888EY12413
dlg1GD4689/CG8888MI13104
dlg1GD4689/dlg15
dlg1GD4689/FsnKG01828
dlg1GD4689/FsnJF01561
dlg1GD4689/Cbp20HMS02428
dlg1GD4689/Pak6
dlg1GD4689/Pak11
dlg1GD4689/PIG-ZHMS01828
dlg1GD4689/Ulp1KK107062
dlg1GD4689/CG5543GD11687
D
FsnGD11383 pairwise interactions
with validation RNAi and mutant lines
Phenotypic score
0
20
40
60
FsnGD11383/Control
FsnGD11383/CG8888EY12413
FsnGD11383/CG8888MI13104
FsnGD11383/dlg15
FsnGD11383/FsnKG01828
FsnGD11383/FsnJF01561
FsnGD11383/Cbp20GD17252
FsnGD11383/Cbp20HMS02428
FsnGD11383/PIG-ZHMS01828
E
PakKK101874 pairwise interactions
with validation RNAi and mutant lines
Phenotypic score
0
20
40
60
PakKK101874/Control
PakKK101874/CG8888EY12413
PakKK101874/CG8888MI13104
PakKK101874/dlg15
PakKK101874/FsnJF01561
PakKK101874/Cbp20GD17252
PakKK101874/Cbp20HMS02428
PakKK101874/Pak11
PakKK101874/PIG-ZHMS01828
PakKK101874/Ulp1KK107062
PakKK101874/CG5543GD11687
F
PIG-ZKK107404 pairwise interactions
with validation RNAi and mutant lines
Phenotypic score
0
20
40
60
PIG-ZKK107404/Control
PIG-ZKK107404/CG8888EY12413
PIG-ZKK107404/CG8888MI13104
PIG-ZKK107404/dlg15
PIG-ZKK107404/dlg1KK116285
PIG-ZKK107404/FsnKG01828
PIG-ZKK107404/FsnJF01561
PIG-ZKK107404/Cbp20GD17252
PIG-ZKK107404/Cbp20HMS02428
PIG-ZKK107404/Pak6
PIG-ZKK107404/Pak11
PIG-ZKK107404/PIG-ZHMS01828
