# Supplementary material for: NCBP2 modulates neurodevelopmental defects of the 3q29 deletion in Drosophila and Xenopus laevis models
Source: PLoS Genet. 2020 Feb 13;16(2):e1008590. doi: 10.1371/journal.pgen.1008590 (PMC7043793; doi:10.1371/journal.pgen.1008590)
Supplement: S3 Table — Blue shaded boxes indicate previously identified phenotypes for individual homologs of 3q29 genes, while “X” marks indicate recapitulated and novel phenotypes identified in our study. Gray-shaded boxes indicate that a homolog was not present in the model organism. Fly phenotypes were obtained from FlyBase [122], X. laevis phenotypes were obtained from Xenbase [120], and mouse knockout model phenotypes were obtained from the Mouse Genome Informatics database [123]. (PDF) [file pgen.1008590.s017.pdf]

3q29 genes	*Drosophila* phenotypes					*X. laevis* phenotypes							Mouse phenotypes						
	Behavior	Development	Lethality	Neuroanatomy	Neurophysiology	Behavioral/ neurological	Cellular	Embryo	Growth/size/body	Mortality/aging	Nervous system	Other	Behavioral/ neurological	Cellular	Embryo	Growth/size/body	Mortality/aging	Nervous system	Other
BDH1 (CG8888)	X	X		X															
CEP19																			
DLG1 (dlg1)	X		X	X															
FBXO45 (Fsn)							X				X	X							
LRRC33																			
MFI2 (Tsf2)		X	X																
NCBP2 (Cbp20)	X	X	X	X			X				X	X							
OSTalpha (CG6836)	X		X																
PAK2 (Pak)	X	X		X							X	X							
PCYT1A (Pcyt2)																			
PIGX (PIG-X)	X	X		X															
PIGZ (PIG-Z)	X			X															
RNF168																			
SENP5 (Ulp1)			X																
SMCO1																			
TCTEX1D2 (CG5359)																			
TFRC																			
TM4SF19																			
UBXN7 (CG8892)																			
WDR53 (CG5543)			X	X															
ZDHHC19 (app)	X			X															
