# Supplementary material for: NCBP2 modulates neurodevelopmental defects of the 3q29 deletion in Drosophila and Xenopus laevis models
Source: PLoS Genet. 2020 Feb 13;16(2):e1008590. doi: 10.1371/journal.pgen.1008590 (PMC7043793; doi:10.1371/journal.pgen.1008590)
Supplement: Attachment — Submitted filename: Girirajan_reviewer_responses_PLOS_Genetics.pdf [file pgen.1008590.s035.pdf]

POINT-BY-POINT RESPONSES TO REVIEWER COMMENTS

RESPONSES TO REVIEWER #1 COMMENTS

1. ***We are concerned about the use of the terms "pathogenicity" and "causative" in several places throughout the manuscript. The American College for Medical Genetics defines criteria for determining the pathogenicity of specific sequence variants for medical genetics. The deletions being discussed in this paper are often already considered pathogenic in patients without the data presented by the authors here. The authors seem to use the term pathogenicity when they mean the potential for underlying mechanism in gene interactions, but fundamentally this paper does not address a disease mechanism therefore this is not demonstrated pathogenicity. We would request that they remove entirely any use or reference of their work in flies using pairwise crosses to denote pathogenicity defined in humans. The first example includes within the abstract "These cellular and neuronal defects were rescued…suggesting that apoptosis is one of several potential biological mechanisms for pathogenicity of the deletion." AND "Overall our study suggests that NCPB2-mediated genetic interactions disrupt apoptosis and cell cycle mechanisms during development, contributing to the neurodevelopmental features of the 3q29 deletion." These statement make too many assumptions about the shared processes between flies and humans. The authors need to scale back these expansive clinical conclusions and simply describe their data. They should remove "pathogenicity" from every instance in this manuscript.***

Response: We agree with the reviewer that the results we observed in *Drosophila* and *X. laevis* models may not have direct translational relevance in humans. To address this comment, we have revised the main text to emphasize our results in the context of cellular and biological mechanisms rather than their clinical relevance. For example, we have removed all instances of the word "pathogenicity" from our manuscript, except when describing "pathogenic CNVs" or "gene pathogenicity metrics" specifically in the human context in the Introduction and Discussion. Below, we highlight a subset of our revisions to the main text, including sections of the Abstract that were mentioned by the reviewer:

Abstract, page 2*: "Despite its importance towards neurodevelopment, the role of individual genes, genetic interactions, and disrupted biological mechanisms underlying the deletion have not been thoroughly characterized. ... These cellular and neuronal defects were rescued with overexpression of the apoptosis inhibitors Diap1 and xiap in both models, suggesting that apoptosis is one of several potential biological mechanisms disrupted by the deletion. ...Overall, our study suggests that NCBP2-mediated genetic interactions within the 3q29 region disrupt apoptosis and cell cycle mechanisms during development."*

Introduction, page 5: *"Here, we used a mechanistic approach to understand the role of individual homologs of 3q29 genes and their interactions towards the cellular processes underlying the deletion. ... Our results support an oligogenic model for the 3q29 deletion, and implicate specific cellular mechanisms disrupted by genes in the deletion region."*

Results, page 7: *"Overall, our data show that multiple conserved homologs of genes in the 3q29 region beyond just dlg1 or Pak are important for Drosophila neurodevelopment, suggesting an oligogenic model for the deletion as opposed to a single causative gene."*

Results, page 9: *"As knockdown fly models for homologs of multiple 3q29 genes showed a variety of neuronal, developmental, and cellular defects, we hypothesized that these genes could interact with each other to further disrupt cellular processes during development.*

Results, page 12: *"These results suggest that apoptosis mediates the cellular defects observed in flies with knockdown of Cbp20 and dlg1, emphasizing its role as a potential mechanism underlying the deletion.*

Discussion, page 16-17: *"Using complementary Drosophila and X. laevis models, we interrogated developmental effects, cellular mechanisms, and genetic interactions of individual homologs of genes within the 3q29 region. ... These data, combined with our findings in Drosophila and X. laevis, implicate multiple genes within the 3q29 region as potential candidates for neurodevelopmental defects. ...Our screening of 161 crosses between pairs of fly homologs of 3q29 genes identified 44 interactions that showed enhanced rough eye phenotypes, suggesting that complex interactions among genes in the 3q29 region could contribute towards developmental defects."*

2. ***Figure 2C: The phenotype scores reported in figure 2C for dlg1 and Cbp20 do not reflect what is reported in other figures. For example, 2C reports an average score of ~40 for Cpb20, while it has an average score of ~30 in figure 3B. This is concerning, and calls into question the inter-experiment accuracy of flynotyper.***

Response: We would like to clarify that the phenotypic scores shown for knockdown of *dlg1* and *Cbp20* in **Figures 2B and 3C** were derived from two different experimental crosses. **Figure 2B** shows phenotypic scores for flies obtained from crosses between RNAi lines of individual fly homologs and lines carrying both *GMR-GAL4* and *UAS-Dicer2* (non-recombined lines), while **Figure 3B** shows data for flies obtained from crosses between recombined lines of individual homologs (i.e. RNAi lines generated to contain *dCad-GFP,GMR-GAL4*, but not *UAS-Dicer2*) and y^1,w^{1118} KK controls (VDRC #60100). We also note that the *Flynotyper* scores included in **Figure S3C** show non-recombined RNAi lines of individual homologs crossed with lines carrying *dCad-GFP,GMR-GAL4* without *UAS-Dicer2*. The variability in phenotypic scores among these experiments could therefore be attributed to differences in the genetic makeup of each fly line. We note that the full genotype compositions of the recombined lines are now presented in **S2 File** (shown below as **Table R1**).

We used recombined RNAi lines of individual homologs crossed with KK controls as experimental controls for the pairwise interaction experiments, as lines with pairwise knockdown of two homologs were generated by crossing the recombined line to a line expressing a second RNAi construct or mutation. Although we mentioned this in the Figure Legends, we apologize to the reviewer that we did not clarify this important point in the Figures themselves. Therefore, we have modified the nomenclature for the controls in the main and Supplementary Figures to

represent recombined lines crossed with controls as "*Cbp20*^{KK109448}*/Control*" instead of "*Cbp20*^{KK109448}", which now solely refers to non-recombined lines. We have also denoted the presence of *UAS-Dicer2* for the RNAi lines shown in **Figure 2C**. Furthermore, we have clarified the use of recombined lines as controls for pairwise interaction experiments in the Results of the manuscript:

Results, page 9: *"We found a significant enhancement in eye phenotypic severity, measured using Flynotyper and validated with a second line when available, for 39 pairwise knockdowns compared with recombined lines crossed with control flies (represented in the figures as Cbp20*^{KK109448}*/Control)."*

Results, page 13: *"We found that 34 pairwise interactions, validated with a second line when available, led to significant increases in eye phenotypes compared with recombined lines for individual homologs of 3q29 genes."*

Table R1. Full genotypes for recombined RNAi lines of homologs of 3q29 genes.

RNAi line	Driver	Genotype
app^{KK108227}	*dCad-GFP,GMR-GAL4*	*y*^{1} *w*^{1118}*;dCad-GFP,GMR-GAL4,P{KK108227}/CyO*
Cbp20^{KK109448}	*dCad-GFP,GMR-GAL4*	*y*^{1} *w*^{1118}*;dCad-GFP,GMR-GAL4,P{KK109448}/CyO*
Cbp20^{KK109448}	*Elav-GAL4*	*y*^{1} *w*^{1118}*,Elav-GAL4; P{KK109448}/CyO*
CG6836^{KK112485}	*dCad-GFP,GMR-GAL4*	*y*^{1} *w*^{1118}*;dCad-GFP,GMR-GAL4,P{KK112485}/CyO*
CG8888^{GD3777}	*dCad-GFP,GMR-GAL4*	*w*^{1118}*;dCad-GFP,GMR-GAL4,P{GD3777}/CyO*
dlg1^{GD4689}	*dCad-GFP,GMR-GAL4*	*w*^{1118}*;dCad-GFP,GMR-GAL4/CyO;P{GD4689}*
Fsn^{GD11383}	*dCad-GFP,GMR-GAL4*	*w*^{1118}*;dCad-GFP,GMR-GAL4/CyO;P{GD11383}*
Pak^{KK101874}	*dCad-GFP,GMR-GAL4*	*y*^{1} *w*^{1118}*;dCad-GFP,GMR-GAL4,P{KK101874}/CyO*
PIG-X^{KK109717}	*dCad-GFP,GMR-GAL4*	*y*^{1} *w*^{1118}*;dCad-GFP,GMR-GAL4,P{KK109717}/CyO*
PIG-Z^{KK107404}	*dCad-GFP,GMR-GAL4*	*y*^{1} *w*^{1118}*;dCad-GFP,GMR-GAL4,P{KK107404}/CyO*

3. ***Figure 2E / Line 1319-1320: "To account for reduced DLG expression in pupal eyes with knockdown of dlg1, images were taken at a higher intensity than control images". All images should be taken with a standardized protocol to allow for accurate comparison.***

Response: We performed DLG staining to visualize the cellular structure of the pupal eye with knockdown of homologs of 3q29 genes. In order to compensate for reduced DLG expression in pupal eyes with knockdown of *dlg1*, *Cbp20/dlg1*, and *dlg1/Diap1*, we used a higher intensity laser setting to obtain these images. We agree with the reviewer that confocal images taken to compare expression intensity or to quantify stained cells (i.e. pH3 and dcp1 staining experiments) require that all images be taken with uniform microscopy settings. However, we only used DLG as a marker to visualize cellular boundaries in the pupal eye, and not for any quantitative analysis or comparison of expression levels with other RNAi lines. We believe that this adjustment would not "*obscure, eliminate, or misrepresent any information*" present in the image, as noted in the *PLOS Genetics* Figures policy.

To address this comment, we have modified both the Methods and Legends for **Figures 2E, 5B** and **6D** to clarify that we did not use the DLG images for any quantitative analysis. If the reviewers or editors raise further concerns regarding this issue, we would also be willing to remove these images from the manuscript.

Methods, page 24: *"As DLG staining was only used to visualize cell boundaries in the pupal eye and not for any expression or quantitative analysis, we increased the laser intensity from 400-490V in control flies to 530-570V in flies with knockdown of dlg1 to account for decreased DLG expression."*

Figure 2 legend, page 41: *"We note that pupal eye images were taken at a higher intensity for lines with knockdown of dlg1 to account for reduced expression of DLG (see Methods), as these images were only for visualization of cell boundaries in the pupal eye and not for any quantitative analysis."*

4. ***Line 231-232: The wording here is somewhat confusing in relation to the figure 2E. Does this not cover the entire area reported in the figure? If not, why was the region "adjacent" to the morphogenic furrow chosen as opposed to the entire developing eye?***

Response: We would like to clarify that we have only quantified cells posterior to the morphogenetic furrow in the larval eye disc for the dcp1, TUNEL, pH3 and BrdU experiments, as correctly represented by the white boxes on the control image in **Figures 2E** and **S2C**. We were primarily interested in detecting changes in cellular processes among differentiating neuronal cells that are located posterior to the morphogenetic furrow in the larval eye disc, a location classically used to examine cellular processes in the developing fly eye (for example, please see Greenwood and Struhl, *Development* 1999; Ma *et al*, *Cell* 1993; Strutt and Mlodzik, *Development* 1997). We have therefore modified the text to clarify that only cells posterior to the morphogenetic furrow were quantified:

Results, page 8*: "To test this, we stained the third instar larval eye discs for select knockdowns of individual homologs of 3q29 genes with anti-pH3 (phospho-Histone H3 (Ser10)) and Drosophila caspase-1 (dcp1), markers for proliferating and apoptotic cells, and quantified the number of cells posterior to the morphogenetic furrow (**S2 Fig.**)."*

5. ***Line 233-234: Please report the p-value for the PIG-Z and dlg1 "trends towards increased pH3-positive cells".***

Response: Although we provided the p-vales for all experimental tests performed in the **S5 File**, we apologize for not clarifying the non-significant trends in the main text as well. The p-values for these tests (Mann-Whitney two-tailed tests with Benjamini-Hochberg correction) are p=0.165 for *PIG-Z*, and 0.353 for *dlg1*. To avoid overstating our conclusions, we have modified this

sentence by removing that the *dlg1* pH3 experiment showed a "*non-significant trend*" and adding the p-value for the *PIG-Z* pH3 experiment:

Results, page 8: *"We observed a significant decrease in pH3-positive cells for CG8888 knockdown flies and trends towards increased pH3-positive cells for PIG-Z (PIGZ) knockdown flies compared with GMR-GAL4 controls (p=0.165) (**Fig. 2F, S4 Fig.**), while knockdown of dlg1 led to significant increases in cells stained with bromodeoxyuridine (BrdU), a marker for replicating cells (**S4 Fig.**)."*

We have also added p-values to the main text to describe non-significant trends for changes in *X. laevis* brain area after knockdown of homologs of 3q29 genes:

Results, page 14: *"We also found that simultaneous knockdown of ncbp2 with fbxo45 caused a significant decrease in forebrain size and a trend towards decreased midbrain size (p=0.093) compared with ncbp2 knockdown (**Fig. 8A-C**). Knockdown of pak2 with ncbp2 showed a similar trend towards decreased forebrain size (p=0.051)."*

6. ***Line 256-258: Please report the nature of the enhancement in phenotypic severity in the eye, such as the distance or angle OD score.***

Response: For our pairwise interaction studies, we determined enhancement of the rough eye phenotype if the phenotypic score for pairwise knockdown was significantly different from the phenotypic scores obtained from recombined lines crossed with controls. Since the main phenotypic scores from *Flynotyper* did not reflect the necrotic patches observed in flies with overexpression of *Dronc*, we further assessed *Flynotyper* subscores for distance and angle ommatidial disorderliness (OD) for these flies, as shown in **Figures S10B-C**. However, we found that the subscores also did not capture the necrotic patches, as *Flynotyper* uses the log sum of these two subscores to calculate the overall phenotypic score (Iyer *et al, G3* 2016). In fact, we observed a strong correlation between the distance and angle OD scores and the main phenotypic score.

We also observed a similar correlation between angle and distance OD scores and overall phenotypic scores for the 170 single-gene and pairwise knockdowns of homologs of 3q29 genes used in the interaction studies (**Figure R1**). Given the strong correlation between the phenotypic scores and OD subscores, we believe that these subscores do not provide any further insight into the enhanced rough eye phenotypes, and we are therefore only showing the overall phenotypic scores for these experiments (**Figure 3B-C, S6 Figure, S7 Figure**).

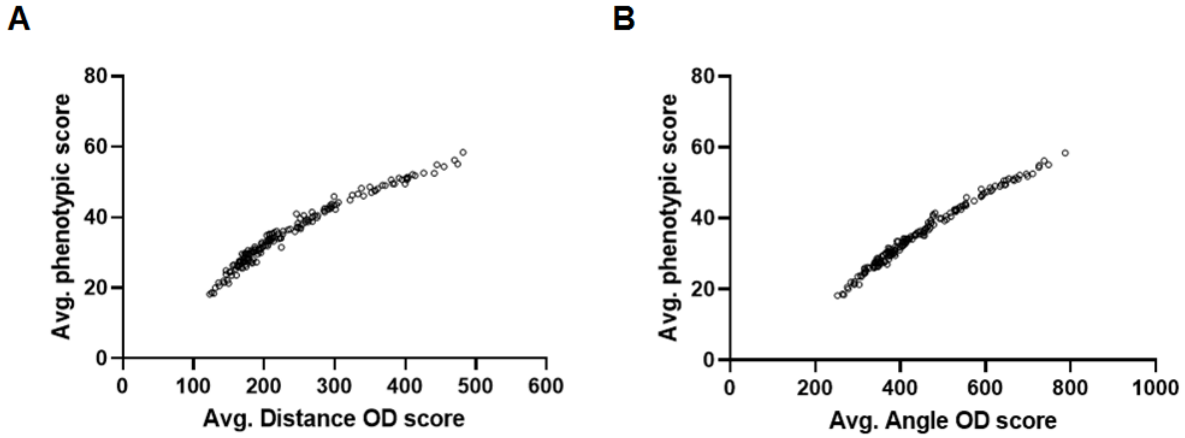

Figure R1. Scatter plots are shown comparing average **(A)** Distance OD (ommatidial disorganization) scores and **(B)** Angle OD scores for 170 single-gene and pairwise knockdown experiments with their respective phenotypic scores from *Flynotyper*. Each individual subscore showed a strong correlation with the main phenotypic score (r=0.9767 for Distance OD and r=0.9910 for Angle OD, $p<0.0001$, Pearson's correlation).

7. ***Figure S4: How are the axon phenotypes scored? What is the difference between mild/moderate/severe?***

Response: We manually scored between 8-20 larval eye disc images per RNAi line or pairwise cross as either "mild", "moderate", or "severe" based on the phenotypic severity of the axon targeting defect (see **Figure R2** for example images). For instance, "*mild*" scores would indicate minor disorganization of the axons in the larval brain; "*moderate*" would indicate partial loss of axon projection, particularly of the R7-R8 axons into the medulla; and "*severe*" would indicate loss of projections for most of the axons at the lamina. Based on the reviewer's query, we have now added these details to the Methods section of the paper, while **S4 Table** (shown below as **Table R2**) displays the scoring for each tested RNAi line or pairwise cross.

Methods, page 24*: "Images stained with anti-chaoptin were manually scored as having either "mild" (minor axon disorganization compared with control), "moderate" (partial loss of axon projection. i.e. loss of R7-R8 projection into the medulla), or "severe" (loss of projections for most axons at the lamina) axon targeting defects."*

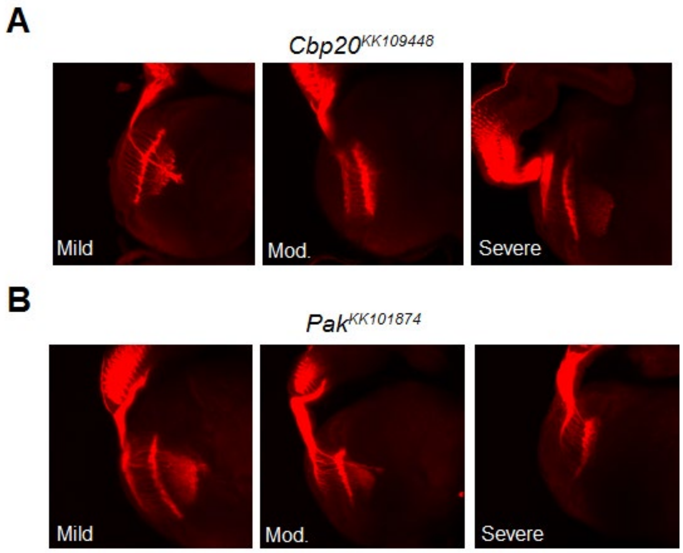

Figure R2. Axon targeting defects with single and pairwise *GMR-GAL4* RNAi knockdown of homologs of 3q29 genes. Individual images of larval eye discs with knockdown of **(A)** *Cbp20* and **(B)** *Pak* were scored as having mild, moderate (Mod.) or severe axon targeting phenotypes.

Table R2. Summary of scoring for phenotypic severity of axon targeting defects upon individual and pairwise knockdown of homologs of 3q29 genes.

**RNAi line**	**Mild axon targeting phenotypes**	**Moderate axon targeting phenotypes**	**Severe axon targeting phenotypes**
$Cbp20^{KK109448}$	4/9	3/9	2/9
$dlg1^{GD4689}$	0/7	2/7	5/7
$Fsn^{GD11383}$	7/20	7/20	6/20
$Pak^{KK101874}$	2/8	4/8	2/8
$Cbp20^{KK109448}$/ $dlg1^{GD4689}$	2/17	8/17	7/17
$Cbp20^{KK109448}$/ $Fsn^{GD11383}$	1/16	4/16	11/16
$Cbp20^{KK109448}$/ *Overexp. Diap1*	5/11	6/11	0/11
$dlg1^{GD4689}$/ *Overexp. Diap1*	1/17	8/17	8/17

Individual larval eye disc images were assigned mild, moderate or severe scores based on the severity of axon projection loss observed in each eye disc. We found that the mild to moderate defects observed with knockdown of *Cbp20* were enhanced with concomitant knockdown of *dlg1* or *Fsn*, while *Diap1* overexpression partially rescued the defects observed with knockdown of *Cbp20* or *dlg1*.

8. ***Figure S10A: The panel displaying the cell organization of animals overexpressing Dronc is missing.***

Response: We acknowledge the missing panel in Figure S10A, and note that this is because of the arrangement of individual images in the figure. We clarify that we did not perform the DLG staining experiments for eyes with overexpression of *Dronc*, and have now further clarified this in the accompanying **S10 Figure** legend.

S10 Figure Legend, page 52*: "Representative brightfield adult eye images (scale bar = 100 µm) and confocal images of larval eye discs (scale bar = 30 µm) stained with anti-dcp1 are shown for flies with GMR-GAL4 overexpression of Diap1 and Dronc, while confocal images of pupal eyes (scale bar = 5 µm) stained with anti-DLG are also shown for flies with overexpression of Diap1."*

9. ***Figure 7B / Line 389-390: Please provide a quantification of the necrotic patches shown in addition to the representative figures.***

Response: We thank the reviewer for this useful suggestion. We have now quantified the area of necrotic patches in adult fly eyes with knockdown of *Cbp20/arm* and *dlg1/arm*, and compared the results with eyes with individual knockdown of *Cbp20*, *dlg1*, and *arm*. We found that flies with pairwise knockdown of *dlg1/arm* showed significantly increased necrotic patches compared to individual knockdown of *dlg1* and *arm* ($p<0.05$, one-tailed Mann-Whitney test with Benjamini-Hochberg correction), while flies with *Cbp20/arm* pairwise knockdown showed increased necrotic patches compared with *Cbp20* knockdown. These data have been added to **Figure S9A** (shown below as **Figure R3**) alongside the results for necrotic patch quantification with pairwise knockdown of homologs of 3q29 genes, and the Results section has been revised accordingly.

Results, page 13: *"Furthermore, knockdown of dlg1 significantly enhanced the ommatidial necrotic patches observed with concomitant knockdown of arm (CTNNB1), while flies with knockdown of Cbp20 and arm also showed increased necrotic patches (**Fig. 7B, S9 Fig.**)."*

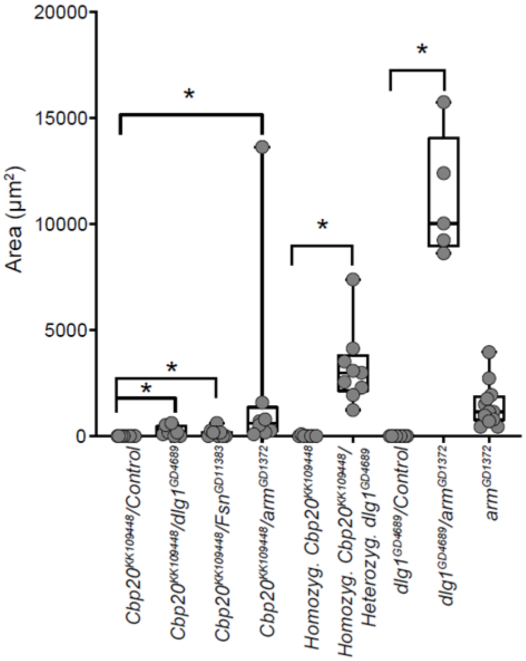

Figure R3. Box plot showing the area of necrotic patches in adult fly eyes with pairwise knockdown of homologs of 3q29 genes (n=5-13, $*p < 0.05$, one-tailed Mann–Whitney test with Benjamini-Hochberg correction). Flies with knockdown of *Cbp20* and *Fsn, dlg1* and *arm* showed enhanced necrotic patches compared with knockdown of *Cbp20*, while homozygous *Cbp20* RNAi and concomitant knockdown of *dlg1* showed increased necrotic patches compared with homozygous *Cbp20* RNAi. Furthermore, flies with pairwise knockdown of *dlg1* and *arm* both showed enhanced necrotic patches compared with individual knockdown of *dlg1* or *arm*. All boxplots indicate median (center line), 25th and 75th percentiles (bounds of box), and minimum and maximum (whiskers), with red dotted lines representing the control median. A list of full genotypes for fly crosses used in these experiments is provided in S2 File.

10. ***Figure 8D: The western blot for apoptosis markers appears to have multiple caspase-3 bands for the fbxo45 knockdown and fbxo45/ncbp2 knockdown, which are absent in other lanes. Is there an explanation of these bands? Is it possible there could be an error the measurement of band intensity due to their presence?***

Response: The antibody that we used for the Western blot experiments (9661S, Cell Signaling Technology) shows two bands for cleaved caspase-3, one at 17kD and one at 19kD (see https://media.cellsignal.com/pdf/9661.pdf for manufacturer documentation; see also examples in He *et al*, *Cancer Res.* 2003 and Zhang *et al*, *J. Neurosci.* 2007). We measured both of these bands when quantifying the band intensity in **Figure 8E**. We have now clarified that there are two bands for cleaved caspase-3 in the main figure legend.

Figure 8 legend, page 45: *"Western blot analysis of X. laevis whole embryos show increased intensity of cleaved caspase-3 bands at 19kD and 17kD with knockdown of homologs of 3q29 genes, including enhanced caspase-3 levels with knockdown of multiple homologs of 3q29 genes and rescued levels with xiap overexpression (red box)."*

RESPONSES TO REVIEWER #2 COMMENTS

Your manuscript is very interesting both in terms of genetic screens and for the identification of candidate genes for the 3q29 region. Nevertheless I have a few questions regarding the validation of the down-regulation of the 3q29 homologs genes.

1. ***You only showed the s2 table for Elav-Gal4 but did you perform the validation of the levels of expression using other GAL4 drivers in drosophila or with morpholinos in Xenopus L? Can you show some validation experiments and in particular have you done it while performing gene interaction study? Were you able to find your gene of interest down-expressed using the RNAseq experiments in fly?***

Response: We thank the reviewer for raising this important point. Our data have several lines of evidence besides the *Elav-GAL4* derived qPCR data, which suggests that our model systems showed reduced expression of homologs of 3q29 genes:

(i) We used samples from *Elav-GAL4* (pan-neuronal) mediated knockdown of homologs of 3q29 genes for performing qPCR on whole fly heads. Although we did not perform qPCR experiments for knockdown of homologs with other *UAS-GAL4* drivers (such as *GMR-GAL4*, bx^{MS1096}*-GAL4* or *da-GAL4*), we observed a range of developmental phenotypes for each individual homolog knocked down with these drivers. For example, 7/14 homologs showed early lethality or wing defects with ubiquitous *da-GAL4* mediated knockdown, while 9/14 homologs showed lethality or rough eye defects with eye-specific *GMR-GAL4* mediated knockdown. Furthermore, each of the 3q29 homologs showed enhanced rough eye defects with concomitant knockdown of at least one other homolog in our pairwise interaction experiments. As we previously showed that *GMR-GAL4* lines produced enough GAL4 to bind two RNAi constructs (Iyer et al, *Nat. Commun*. 2018), these results suggest that each homolog was knocked down in the fly eye in our interaction studies.

(ii) Following the reviewer's useful suggestion, we examined the expression of homologs of 3q29 genes after *Elav-GAL4* mediated knockdown in our RNA-seq experiments (performed using three biological replicates). The expression of each homolog knocked down in each RNA-seq experiment was compared with the expression obtained from VDRC GD or KK controls using the edgeR software (Robinson *et al*, *Bioinformatics* 2010). Overall, we found that 5/6 sets of RNA-seq experiments showed significant (false discovery rate<0.05) knockdown of their respective homologs, including for both pairwise knockdown lines tested (**Table R3**). The exception was for *dlg1*, which showed a non-significant 12% decrease in expression compared with 37% decrease in expression observed with qPCR data. We note that tissue-specific knockdown of $dlg1^{GD4689}$ showed several developmental phenotypes in our study, including significant climbing defects in adult flies with *Elav-GAL4* mediated knockdown of $dlg1^{GD4689}$. Furthermore, this RNAi line ($dlg1^{GD4689}$) has been used extensively to model knockdown of *dlg1* in flies (for example, see Willecke *et al, Oncogene* 2011; Mauri *et al, Curr. Biol*. 2014; Katheder *et al, Nature* 2017). This suggests that the RNA-Seq results could be due to variability in

the RNA-Seq experiment, and could also explain the differences in expression values between qPCR and RNA-Seq data observed in the other five lines.

Table R3. Expression of homologs of 3q29 genes in RNA-Seq and qPCR experiments.

Fly gene	qPCR % expression	RNA-Seq experiment	RNA-Seq % expression	RNA-Seq FDR
Cbp20	43.90%	*Cbp20^{KK109448}*	36.49%	4.25E-10
		Cbp20^{KK109448}/dlg1^{GD4689}	40.65%	1.76E-09
		Cbp20^{KK109448}/Fsn^{GD11383}	42.58%	2.63E-09
dlg1	62.69%	*dlg1^{GD4689}*	87.87%	0.265
		Cbp20^{KK109448}/dlg1^{GD4689}	78.41%	0.013
Fsn	55.23%	*Fsn^{GD11383}*	67.51%	2.35E-05
		Cbp20^{KK109448}/Fsn^{GD11383}	69.30%	6.03E-05
Pak	40.21%	*Pak^{KK101874}*	75.33%	0.017

(iii) We would like to note that we also previously performed qPCR validation for morpholino knockdown of individual 3q29 homologs in *X. laevis* models, presented in **Figure S12B** (shown below as **Figure R4A**). Each morpholino showed significant knockdown of gene expression compared with controls. Furthermore, each individual *X. laevis* homolog showed defects in eye area upon morpholino-induced knockdown, which were rescued with mRNA overexpression of each homolog (**Figure S13C**, shown below as **Figure R4B**).

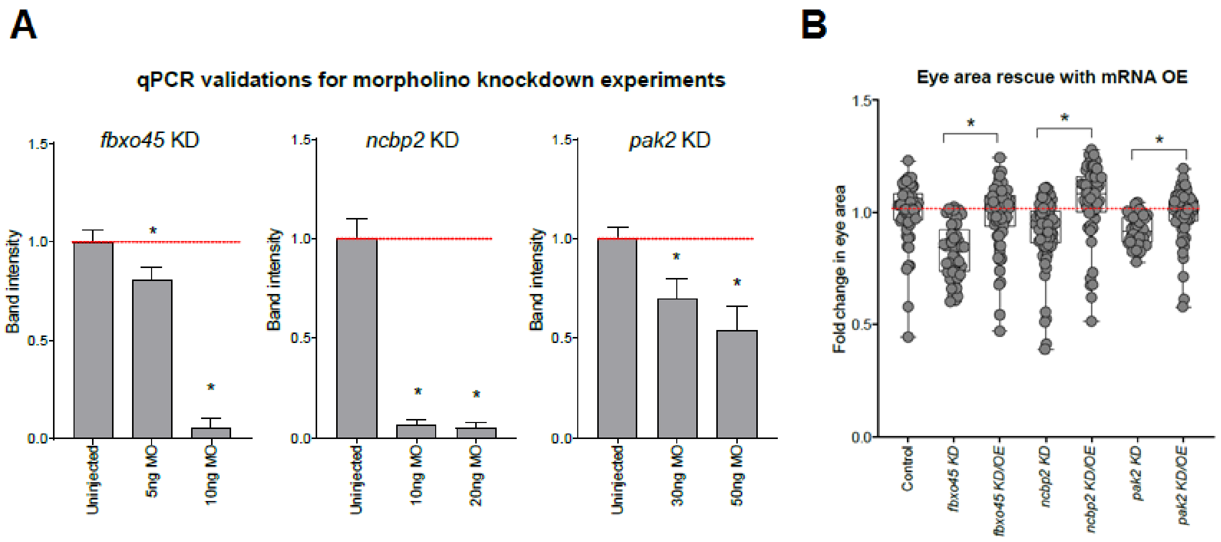

Figure R4. Validation of morpholino knockdown of *X. laevis* homologs of 3q29 genes. **(A)** Quantification of expression for homologs of 3q29 genes at different MO concentrations, as measured by band intensity ratio to *ODC1* controls (n=3 replicates, *p<0.05, two-tailed Welch's T-test with Benjamini-Hochberg correction). **(B)** Box plot of eye area in *X. laevis* models with knockdown of homologs of 3q29 genes and overexpression of mRNA for homologs of 3q29 genes, normalized to controls (n = 56–63, *p < 0.05, two-tailed Welch's T-test with Benjamini-Hochberg correction). Boxplots indicate median (center line), 25th and 75th percentiles (bounds of box), and minimum and maximum (whiskers), with red dotted lines representing the control

median. The data shown for the eye area experiments represent pooled results of three experimental batches, and were normalized to the respective controls from each batch.

2. ***It is of particular importance because as you stated in mouse heterozygous animals for Dlg1 and Pak2 do not show any phenotype.***

Response: We apologize to the reviewer for not clarifying this important point in the text. We would like to clarify that mice with heterozygous knockdown of *Dlg1* and *Pak* showed several neurodevelopmental phenotypes when compared with control mice, but that they did not recapitulate the full range of phenotypes observed in mouse models of the entire deletion (**S10 Table**, shown below as **Figure R5**). For example, the *Pak2*$^{+/-}$ knockdown mouse described by Wang and colleagues showed several behavioral defects, including increased self-grooming and decreased social interaction, as well as decreased synaptic density and potentiation (Wang *et al, Cell Rep*. 2018). Furthermore, *Dlg1*$^{+/-}$ mice showed increased amphetamine-induced locomotor activity compared with controls (Rutkowski *et al*, *Mol. Psych.* 2019), and may exhibit additional phenotypes after further testing. However, neither *Pak2*$^{+/-}$ nor *Dlg1*$^{+/-}$ recapitulated the key phenotypes of the deletion mice, including decreased brain volume and startle response defects. Thus, we concluded that other genes within the 3q29 region besides *Pak2* and *Dlg1* contribute to the deletion phenotypes, mirroring our results which show that knockdown of nearly all homologs of genes in the region contribute towards neuronal phenotypes in the fly eye. This model differs from other CNVs where knockdown of a single gene is responsible for nearly all of the deletion phenotypes, such as loss of *RAI1* within chromosome 17p11.2 for Smith-Magenis syndrome (Girirajan *et al*, *J. Med. Genet.* 2005).

We have modified the Discussion to clarify that *Pak2* and *Dlg1* heterozygous mice showed neuronal phenotypes compared with controls, but did not show the same phenotypes as those observed in the deletion mice:

Discussion, page 16*: "Notably, although Dlg1*$^{+/-}$ *or Pak2*$^{+/-}$ *mice showed a range of neuronal phenotypes compared with control mice, they did not recapitulate the major developmental and behavioral features observed in mouse models of the entire deletion [14,15,72], suggesting that the deletion phenotypes are contingent upon haploinsufficiency of multiple genes in the region* ***(S10 Table)****."*

**3q29 deletion mouse models**	*B6J.Del16* $^{+/Bdh1\text{-}Tfrc}$ *(Baba et al.)*	*B6N.Del16* $^{+/Bdh1\text{-}Tfrc}$ *(Rutkowski et al.)*	*B6N.Dlg1* $^{+/-}$ *(Rutkowski et al.)*	*Pak2* $^{+/-}$ *(Wang et al.)*
Weight	Decreased	Decreased	No phenotype	Not tested
Brain size	Decreased	Decreased	Not tested	No phenotype
Locomotor activity	No phenotype	No phenotype	No phenotype	No phenotype
Amphetamine-induced locomotor activity	Not tested	Increased	Increased	Not tested
Anxiety (elevated plus maze or open field)	Not tested	No phenotype	No phenotype	No phenotype
Spatial learning and memory (water maze)	Not tested	Decreased	No phenotype	No phenotype
Acoustic startle response	Increased	Increased	No phenotype	No phenotype
Prepulse inhibition/sensorimotor gating	Decreased	No phenotype	No phenotype	No phenotype
Startle response w/risperidone	Rescued	Not tested	Not tested	Not tested
Marble burying	Not tested	No phenotype	No phenotype	Increased
Self-grooming	Increased	Not tested	Not tested	Increased
Social interaction (free or 3-chamber)	Decreased	Decreased	No phenotype	Decreased
Fear conditioning (context)	Decreased	No phenotype	No phenotype	Not tested
Auditory excitatory neuron activity	Increased	Not tested	Not tested	Not tested
Parvalbumin neuronal count	Decreased	Not tested	Not tested	Not tested
Dendritic spine density	Not tested	Not tested	Not tested	Decreased
Long-term potentiation	Not tested	Not tested	Not tested	Decreased
Synaptic density	Not tested	Not tested	Not tested	Decreased
Neuronal migration	Not tested	Not tested	Not tested	Decreased
Transcriptome	Immediate early signaling genes	Not tested	Not tested	Post-synaptic density, cytoskeleton, channel activity

Figure R5. Comparison of mice with heterozygous deletion of the syntenic 3q29 region with heterozygous knockout mouse models for *Dlg1*and *Pak2*. Blue shaded boxes indicate phenotypes observed in the knockout models, while gray-shaded boxes indicate a phenotype that was not tested in the knockout model. Neither *Dlg1*$^{+/-}$ nor *Pak2*$^{+/-}$ knockout mice recapitulate the body and brain weight, spatial learning and memory, or acoustic startle defects observed in the deletion mouse models.
